# Dual relevance of *EIF2S1* in acute myeloid leukemia: linking poor prognosis to immune-metabolic states and leukemic cell fitness

**DOI:** 10.3389/fonc.2026.1922910

**Published:** 2026-08-05

**Authors:** Xiaoying Hong, Yingying Huang, Wei Wu, Xiandong Jiang, Yanfeng Lin, Yan Xue, Donghong Lin

**Affiliations:** 1 Department of Laboratory Medicine, The School of Medical Technology and Engineering, Fujian Medical University, Fuzhou, China; 2 Key Laboratory of Clinical Laboratory Technology for Precision Medicine (Fujian Medical University), Fujian Province University, Fuzhou, China; 3 Medical Technology Experimental Teaching Center, The School of Medical Technology and Engineering, Fujian Medical University, Fuzhou, China

**Keywords:** acute myeloid leukemia, *EIF2S1*, immune micro-environment, metabolic remodeling, prognostic biomarker, single-cell RNA sequencing

## Abstract

**Background:**

Most transcriptomic studies in acute myeloid leukemia (AML) have focused on transcriptional regulation, whereas the clinical and biological relevance of translation initiation factors remains insufficiently defined. Eukaryotic translation initiation factor 2 subunit alpha (*EIF2S1*) is a key regulator of translation initiation, but its prognostic significance and association with AML remain unclear.

**Methods:**

Bulk RNA-seq data from the TCGA-LAML and GTEx cohorts were analyzed to evaluate *EIF2S1* expression, prognostic value, associated biological programs, ssGSEA-derived immune-cell-associated signature scores, and quanTIseq-estimated immune-cell proportions. Single-cell RNA sequencing data were further examined to characterize the cellular distribution of *EIF2S1* and subset-specific immune and metabolic transcriptional features. Finally, siRNA-mediated *EIF2S1* knockdown assays were performed in THP-1 monocytic AML cells to assess its functional relevance.

**Results:**

*EIF2S1* was significantly upregulated in AML and independently associated with shorter overall survival in the TCGA-LAML cohort. High *EIF2S1* expression was linked to translation initiation-related programs and immune- and myeloid-lineage-related transcriptional features, including stronger myeloid-lineage signals, weaker cytotoxic T-cell-related signatures, and increased expression of immune checkpoint and immunoregulatory genes, including *LGALS9* and *TGFB1*. Single-cell analysis localized *EIF2S1* enrichment primarily to monocyte-like subsets and associated it with mitochondrial metabolic and innate immune transcriptional programs. Functionally, *EIF2S1* knockdown induced G0/G1 cell-cycle arrest, suppressed THP-1 cell proliferation, migration, and invasion, and promoted apoptosis.

**Conclusions:**

*EIF2S1* is associated with adverse prognosis, translation initiation-related programs, and monocyte-like immune-metabolic transcriptional states in AML. Functional findings further suggest that *EIF2S1* contributes to leukemic cell fitness. These results support *EIF2S1* as a potential therapeutic target requiring further experimental and clinical validation.

## 1 Introduction

Acute myeloid leukemia (AML) is a highly heterogeneous hematologic malignancy that continues to be associated with poor clinical outcomes (1). Extensive genomic and transcriptomic studies have identified recurrent mutations, aberrant signaling pathways, and distinct molecular subtypes in AML; however, these alterations do not fully explain the biological complexity and clinical variability of the disease (2, 3). These limitations highlight the need to investigate additional regulatory layers beyond transcription.

Translation initiation represents the rate-limiting step of protein synthesis, and its dysregulation is a fundamental driver in the pathogenesis of various malignancies, including hematopoietic tumors (4, 5). Eukaryotic translation initiation factor 2 subunit alpha (*EIF2S1*) is a central component of the translation initiation machinery that participates in the delivery of initiator methionyl-tRNA to the ribosome (6). While burgeoning evidence in solid tumors suggests that *EIF2S1* activation supports cell survival, metabolic reprogramming, and chemoresistance under microenvironmental stress (7, 8). Importantly, total *EIF2S1* expression and Ser51 phosphorylation of eIF2α represent related but distinct regulatory layers: total expression reflects the abundance of a translation-initiation component, whereas Ser51 phosphorylation is a key regulator of integrated stress response activity and translational output. Thus, *EIF2S1* expression should not be interpreted as equivalent to its phosphorylation status or pathway activity (9, 10). However, the clinical significance and biological relevance of *EIF2S1* expression in AML remain insufficiently characterized.

The bone marrow microenvironment (BMM) of AML is a unique immunosuppressive microenvironment characterized by polarized M2-like macrophages and a depleted T-cell population (11–13). This malignant cellular subset must finely regulate its translational output to balance high metabolic demands with the continuous secretion of potent immunomodulatory factors (14, 15). Immunomodulatory factors secreted by tumor cells are highly dependent on fine-tuning at the translational level (16). As a core component of the translation initiation machinery, *EIF2S1* may be involved in linking translational control with cellular stress responses, metabolic adaptation, and immune-related transcriptional programs (17, 18). However, the cell-type-specific distribution of *EIF2S1* and its associations with the AML immune-metabolic landscape remain unclear.

Accordingly, this study investigated the dual clinical and biological relevance of *EIF2S1* in AML. Bulk RNA-seq data from TCGA-LAML and GTEx were used to evaluate *EIF2S1* expression, prognostic significance, and associated molecular programs. ssGSEA was used to quantify relative immune-cell-associated signature scores, whereas quanTIseq was used to computationally estimate immune-cell proportions. Single-cell RNA-sequencing analysis was performed to characterize the cellular distribution of *EIF2S1* and its associations with immune and metabolic transcriptional states. Finally, we performed *EIF2S1* loss-of-function assays in the monocytic AML cell line THP-1, selected in view of the monocyte-like states identified by single-cell analysis, to evaluate its contribution to leukemic cell fitness, including proliferative capacity, cell-cycle progression, migration, and invasion. Collectively, this study investigated whether *EIF2S1* serves as a prognostic marker associated with monocyte-related immune-metabolic states and contributes to leukemic cell fitness.

## 2 Materials and methods

### 2.1 Data acquisition

Bulk RNA sequencing (RNA-seq) expression profiles and matched clinical follow-up data for The Cancer Genome Atlas acute myeloid leukemia cohort (TCGA-LAML) were downloaded from the UCSC Xena platform. Normal whole-blood samples were obtained from the Genotype-Tissue Expression project (GTEx); samples without clinical information were excluded. TCGA-LAML and GTEx expression data were obtained from the UCSC Xena Toil RNA-seq recompute compendium, in which both datasets were processed using a unified computational pipeline and provided as RSEM-normalized transcripts per million (TPM) values. Expression values were transformed as log_2_(TPM + 1) before analysis. Risk stratification was assigned according to European LeukemiaNet (ELN) criteria (19). Single-cell RNA sequencing (scRNA-seq) data were obtained from Gene Expression Omnibus (GEO; GSE154109), including 8 AML patients and 4 healthy controls.

### 2.2 Bulk expression, survival, and co-expression analyses

*EIF2S1* expression between AML and controls was compared using the Mann-Whitney U test. Patients were dichotomized into *EIF2S1*-high and *EIF2S1*-low groups using the median expression as the cutoff. Overall survival (OS) was analyzed by Kaplan-Meier method and compared by log-rank test. Cox proportional hazards models were fitted using the R survival package for univariate and multivariate analyses to evaluate the independent association of *EIF2S1* with OS. Genome-wide co-expression with *EIF2S1* was assessed using Spearman correlation in the TCGA-LAML cohort.

### 2.3 Protein-protein interaction network

A protein-protein interaction (PPI) network for EIF2S1 was generated using STRING database (20) with a high-confidence threshold (overall score ≥0.900). Network visualization was performed using circlize to generate Circos plots.

### 2.4 Differential expression and enrichment analyses

Differential expression analysis between the *EIF2S1*-high and *EIF2S1*-low groups was performed using the limma package. *EIF2S1*-high and *EIF2S1*-low cases were defined using the cohort median. A linear model was fitted to the log_2_(TPM + 1)-transformed expression matrix, and empirical Bayes moderation was applied to obtain moderated t-statistics. Genes with an absolute log_2_ fold change greater than 1 and a Benjamini-Hochberg-adjusted *p* value below 0.05 were considered differentially expressed. Gene Ontology (GO) and Kyoto Encyclopedia of Genes and Genomes (KEGG) enrichment analyses were performed using clusterProfiler (21).

### 2.5 Assessment of immune-cell-related transcriptional features

Immune-cell-related transcriptional features were assessed using two complementary computational approaches. Single-sample gene set enrichment analysis (ssGSEA), implemented in the GSVA package, was used to calculate relative enrichment scores for predefined immune-cell-associated genesignatures (22). quanTIseq deconvolution was used to computationally estimate the relative proportions of predefined immune-cell populations (23, 24). ssGSEA scores and quanTIseq estimates were interpreted as computationally inferred features rather than direct measurements of immune-cell infiltration. To assess potential confounding by monocytic differentiation, sensitivity analyses were performed after excluding FAB M4/M5 cases; detailed procedures are provided in **Supplementary Methods 2**.

### 2.6 scRNA-seq processing and downstream analyses

scRNA-seq data were analyzed using Seurat (25). Low-quality cells and putative doublets were removed by stringent filtering: cells with fewer than 200 or more than 5,000 detected genes (nFeature_RNA), or a total UMI count (nCount_RNA) exceeding 40,000, were excluded. We also removed cells with mitochondrial gene content (percent.mt) > 5%, hemoglobin gene expression (percent.hb) > 0.1%, or ribosome gene expression (percent.ribo) > 30%. After quality control, 3,346 cells were retained for downstream analysis. Data were normalized using SCTransform, followed by principal component analysis (PCA). Batch effects were corrected using Harmony (26) based on the first 22 principal components. The first 22 Harmony dimensions were used for nearest-neighbor graph construction and UMAP visualization, with n.neighbors = 30 and min.dist = 0.3. Louvain clustering was performed using the Seurat FindClusters function at a resolution of 2, yielding 23 clusters. Clusters 0–22 contained 344, 288, 286, 246, 233, 215, 201, 200, 172, 157, 156, 145, 141, 135, 107, 101, 62, 34, 27, 26, 25, 23, and 22 cells, respectively. Cell types were annotated using canonical lineage markers curated in CellMarker 2.0 (27). The candidate marker panels included monocytes (CD14, LYZ, S100A8, S100A9, VCAN, and FCGR3A), CD8^+^ effector memory RA cells (CD3D, CD8A, GZMA, GZMB, PRF1, NKG7, and KLRG1), plasmacytoid dendritic cells (LILRA4, CLEC4C, IL3RA, IRF7, and TCF4), naive B cells (CD79A, MS4A1, IGHD, TCL1A, FCER2, and IL4R), myeloid dendritic cells (CD1C, FCER1A, CLEC10A, HLA-DQA1, and HLA-DQB1), class-switched mature B cells (CD79A, MS4A1, CD27, IGHG1, and IGHA1), and common myeloid progenitors (CD34, KIT, FLT3, IL3RA, CSF1R, and GATA2). The final annotated populations comprised 1,622 monocytes, 825 CD8^+^ effector memory RA cells, 698 naive B cells, 141 common myeloid progenitors, and 60 class-switched mature B cells. We divided the monocyte subsets into an *EIF2S1* positive (detected expression > 0) group and an *EIF2S1* negative (detected expression = 0) group. DEGs were identified using the Wilcoxon rank-sum test, followed by GO and KEGG enrichment analyses.

### 2.7 Cell culture and siRNA-mediated *EIF2S1* knockdown

The human acute monocytic leukemia cell line THP-1 (Cat. No. CL-0233) was purchased from Wuhan Procell Biotechnology Co., Ltd. THP-1 cells were selected as a monocytic AML model because the single-cell analysis associated *EIF2S1* expression with monocyte-like leukemic cell states. The experiments were intended to provide initial functional validation in this specific cellular context. Cells were cultured in RPMI-1640 supplemented with 10% fetal bovine serum (FBS; Gibco) and 1% penicillin–streptomycin at 37 °C with 5% CO_2_. Three *EIF2S1*-targeting small interfering RNAs (*siRNAs*: *si1#, si2#, si3#*) and a negative control (*si-NC*) were used. The siRNA sequences targeting *EIF2S1* were as follows: negative control sense strand: 5’-UUCUCCGAACGUGUCACGUTT-3’, antisense strand: 5’-ACGUGACACGUU CGGAGAATT-3’; siRNA-1 sense strand: 5’-GGCUUGUUAUGGUU AUGAATT-3’, antisense strand: 5’-UUCAUAACCAUAACAAGCCTT-3’; siRNA-2 sense strand: 5’-GUCAAGCUAUGGCUGUUAUTT-3’, antisense strand: 5’-AUAACAGCCAUA GCUUGACTT-3’. siRNA-3 sense strand: 5’-GCUUGCU GGAAUACAACAATT-3’, antisense strand:5’-UUGUUGUAUUCCAGCAAGCTT-3’. Cells were transfected with siRNA-Mate SUS according to the manufacturer’s protocol and harvested 48–72 h post-transfection. Knockdown efficiency was assessed at mRNA and protein levels by real-time quantitative PCR (RT-qPCR) and Western blotting to select the optimal siRNA for functional assays.

### 2.8 RT-qPCR and Western blotting

Total RNA was extracted using TRIzol (Invitrogen) and reverse-transcribed to cDNA. RT-qPCR was performed using Hieff^®^ qPCR SYBR^®^ Green Master Mix (Low ROX); β-actin was used as the internal control. The primer sequences were as follows: β-actin: Forward: GACAGGATGCAGAAGGAGAT;Reverse: GAGGCCAGGATGGAGC;*EIF2S1*:Forward: TGGTGAATGTCAGATCCATTGC;Reverse: TAGAACGGATACGCCTTCTGG. For Western blotting, total protein was extracted using RIPA buffer with protease inhibitors, quantified by BCA assay, separated by SDS-PAGE, and transferred to PVDF membranes. Membranes were blocked with 5% non-fat milk, incubated with primary antibodies (anti-EIF2S1 1:2,000; anti-β-actin 1:2,000) overnight at 4 °C, followed by HRP-conjugated secondary antibodies for 1 h at room temperature. Signals were detected using ECL.

### 2.9 Functional assays

Cell proliferation was measured by Cell Counting Kit-8 (CCK-8; Cat. No. K1018) at 0, 24, 48, 72 and 96 h, with absorbance read at 450 nm after 4 h incubation. Cell-cycle distribution was determined by propidium iodide (PI) staining with RNase (Cat. No. K2263) and analyzed by flow cytometry (FongCyte). Apoptosis was assessed using an Annexin V**-**FITC/PI apoptosis detection kit (Cat. No. K2003) according to the manufacturer’s instructions, followed by flow-cytometric analysis. Migration and invasion were assessed using Transwell assays; invasion inserts were pre-coated with Matrigel (Cat. No.C231001). After 24 h, migrated/invaded cells were fixed, stained with 0.1% crystal violet, and quantified in randomly selected fields.

### 2.10 Statistical analysis

All analyses were performed in R (v4.5.2) and GraphPad Prism (v9.5). Quantitative data are presented as the mean ± standard deviation (SD) of three independent biological experiments. Because only three biological replicates were available per condition, formal normality testing was not performed. qRT-PCR, immunoblot densitometry, migration, invasion, and apoptosis data were analyzed using one-way ANOVA followed by Dunnett’s multiple-comparison test. Time-course CCK-8 data and cell-cycle distributions were analyzed using two-way ANOVA followed by Dunnett’s multiple-comparison test. All tests were two-sided, and P < 0.05 was considered statistically significant. *p* < 0.05 was considered statistically significant (*p < 0.05; **p < 0.01; ***p < 0.001; ****p < 0.0001).

## 3 Results

### 3.1 *EIF2S1* is significantly overexpressed in AML and is positively correlated with poor patient prognosis

**Figure 1:**
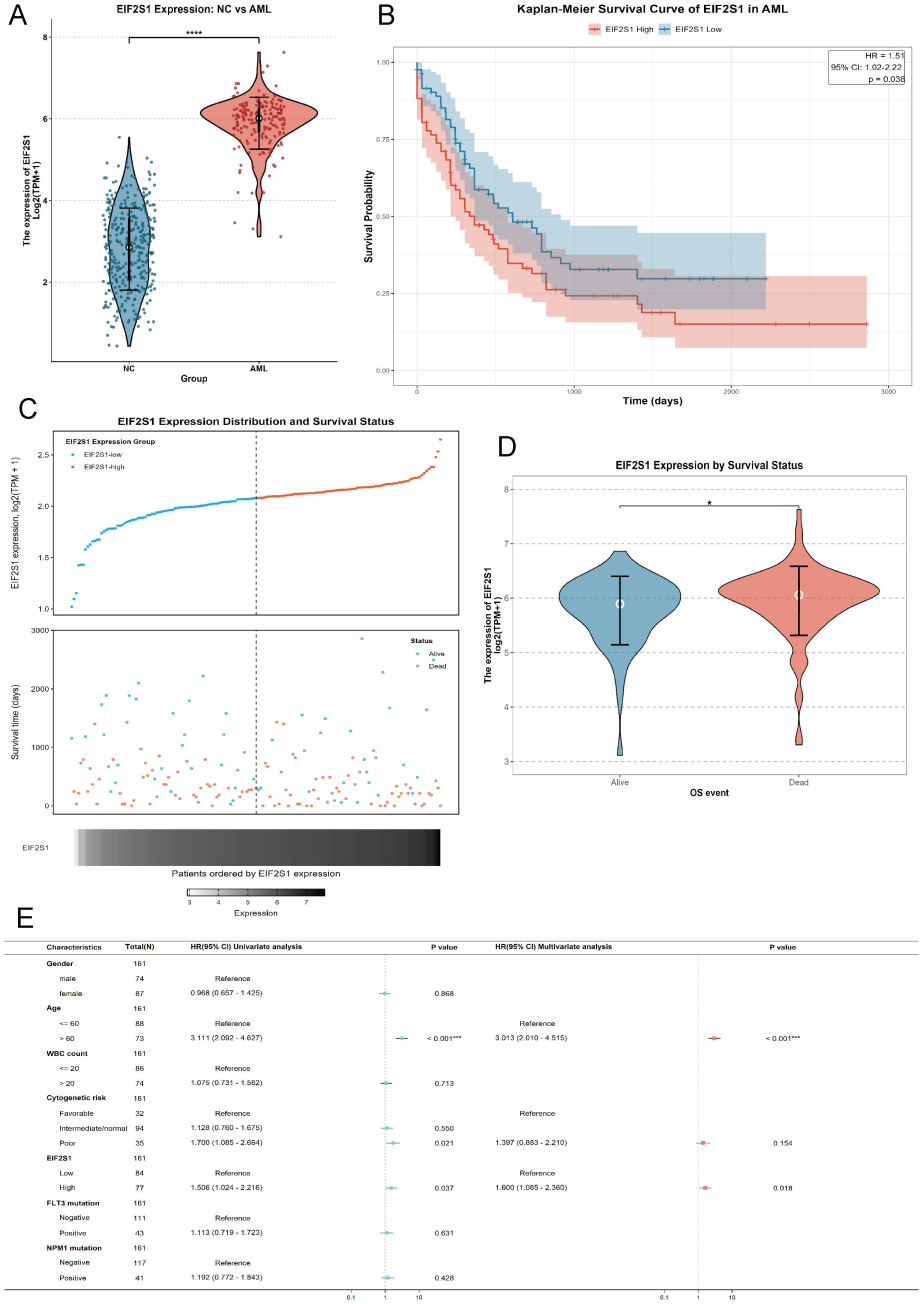
*EIF2S1* expression and prognostic significance in AML. **(A)** *EIF2S1* expression in normal controls (n = 337) and AML samples (n = 173). Groups were compared using a two-sided Wilcoxon rank-sum test (*p* = 6.75 × 10^-74^). **(B)** Kaplan–Meier analysis of overall survival according to the median *EIF2S1* expression level. The *EIF2S1*-low group included 84 patients and 48 deaths, and the *EIF2S1*-high group included 77 patients and 56 deaths. Survival curves were compared using a two-sided log-rank test (*p* = 0.03785); the hazard ratio was estimated using univariable Cox regression. **(C)** Distribution of *EIF2S1* expression and survival status among patients with complete survival information (n = 161; 104 deaths), ordered by *EIF2S1* expression. **(D)** *EIF2S1* expression in surviving (n = 58) and deceased (n = 115) patients. Groups were compared using a two-sided Wilcoxon rank-sum test (*p* = 0.04085). **(E)** Univariable and multivariable Cox proportional hazards analyses of overall survival.

To investigate the clinical significance of *EIF2S1* in AML, we first compared its expression levels between AML patients and normal controls using public databases (TCGA and GTEx). Differential expression analysis revealed that *EIF2S1* was significantly overexpressed in AML patients compared to normal (**Figure 1A**; *p* < 0.0001). To reduce the potential influence of cross-project differences between TCGA-LAML and GTEx, *EIF2S1* expression was independently evaluated in the GSE30029 cohort using cell-type-matched CD34-positive samples. *EIF2S1* expression was significantly higher in AML CD34-positive cells than in normal bone marrow CD34-positive cells (n = 46 versus n = 31; *p* = 0.005913; **Supplementary Figure 1**), supporting the reproducibility of *EIF2S1* upregulation in AML.

Subsequently, Kaplan-Meier analysis subsequently showed that patients in the *EIF2S1*-high group had significantly shorter overall survival than those in the *EIF2S1*-low group (**Figure 1B**; HR = 1.51, 95% CI: 1.02–2.22, *p* = 0.038). Consistently, the distribution of survival outcomes indicated a survival advantage in patients with lower *EIF2S1* expression (**Figures 1C, D**). Univariable Cox regression analysis showed that age >60 years, poor cytogenetic risk, and high *EIF2S1* expression were associated with adverse overall survival. After adjustment for sex, age, white blood cell count, cytogenetic risk, FLT3 mutation status, and NPM1 mutation status, high *EIF2S1* expression remained significantly associated with an increased risk of death (HR = 1.600, 95% CI: 1.085–2.360, *p* = 0.018; **Figure 1E**). The prognostic association of *EIF2S1* was further evaluated in the independent Beat AML 2.0 cohort. Higher continuous *EIF2S1* expression was significantly associated with an increased risk of death after multivariable adjustment (adjusted HR per 1-SD increase = 1.222, 95% CI: 1.032–1.447, *p* = 0.020; **Supplementary Figure 2**). In the complementary dichotomized multivariable analysis, the *EIF2S1*-high group also showed a higher adjusted mortality risk (adjusted HR = 1.37, 95% CI: 1.01–1.86, *p* = 0.040; **Supplementary Figure 3**).

As a complementary analysis in the TCGA-LAML cohort, *EIF2S1* was also modeled as a continuous standardized variable. Each one-standard-deviation increase in *EIF2S1* expression was significantly associated with a higher risk of death after adjustment for sex, age, white blood cell count, cytogenetic risk, FLT3 mutation status, and NPM1 mutation status (adjusted HR = 1.476, 95% CI: 1.110–1.962, *p* = 0.007; **Supplementary Figure 4**). Collectively, these findings support *EIF2S1* as a candidate prognostic marker associated with adverse survival outcomes in AML.

### 3.2 EIF2S1 is co-expressed with translation initiation components in AML

**Figure 2:**
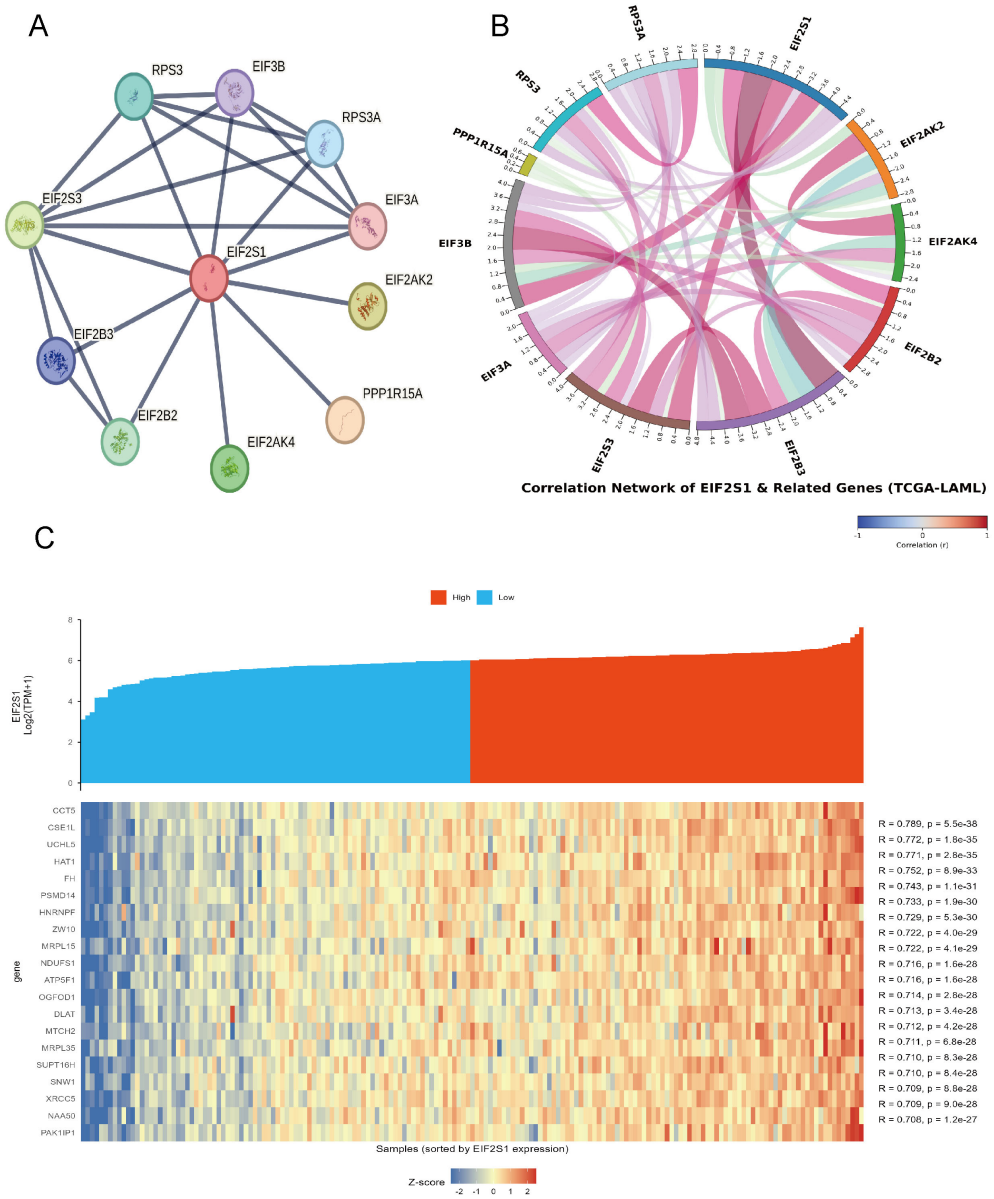
PPI network and highly correlated genes of EIF2S1. **(A)** PPI network of *EIF2S1* and its top 10 interacting proteins, derived from the STRING database (overall score ≥ 0.900, highest confidence). **(B)** Circos plot showing the interactions between *EIF2S1* and the genes encoding its interacting proteins. **(C)** Correlation heatmap between *EIF2S1* expression and the 20 most correlated genes in the TCGA-AML cohort.

To elucidate the molecular interactome landscape of EIF2S1 in AML, a high-confidence PPI network (combined score ≥ 0.900) was first constructed using the STRING database. EIF2S1 was connected to several components of the translation initiation machinery, including EIF2S3, EIF2B2, EIF2B3, EIF3A, and EIF3B, as well as the ribosomal proteins RPS3 and RPS3A (**Figure 2A**). Correlation analysis in the TCGA-LAML cohort further revealed coordinated expression among these translation-related genes (**Figure 2B**).

To define the broader transcriptional context associated with EIF2S1, AML samples were ordered according to EIF2S1 expression, and the top 20 EIF2S1-correlated genes were visualized in a heatmap (**Figure 2C**; *p* < 0.001). All selected genes were positively correlated with EIF2S1, with correlation coefficients ranging from 0.708 to 0.789. Their expression progressively increased across samples with higher EIF2S1 levels, indicating that EIF2S1 upregulation occurs within a coordinated transcriptional program rather than as an isolated expression change. Collectively, these findings place EIF2S1 within a translation initiation-associated molecular network in AML.

### 3.3 *EIF2S1-*high AML exhibits distinct immune and metabolic transcriptional programs

**Figure 3:**
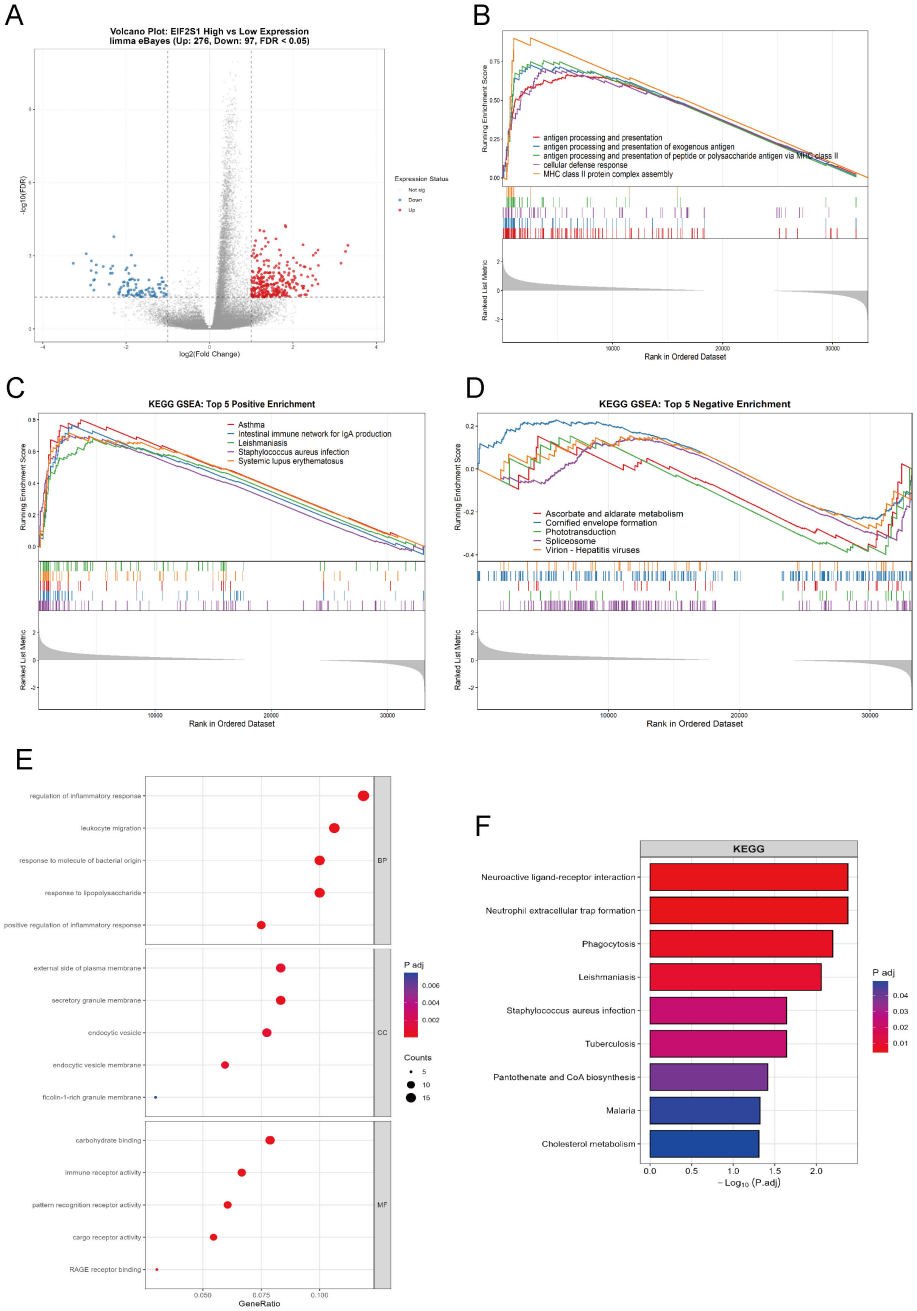
Differential expression and functional enrichment analyses associated with *EIF2S1* expression in AML. **(A)** Volcano plot showing differentially expressed genes between the *EIF2S1*-high and *EIF2S1*-low groups, identified using limma with empirical Bayes moderation. Genes with |log_2_ fold change| > 1 and Benjamini–Hochberg-adjusted *p* < 0.05 were considered significant. **(B)** Ranked-list GSEA showing the top positively enriched gene sets in the *EIF2S1*-high group. **(C)** KEGG GSEA showing the top pathways positively enriched in the *EIF2S1*-high group. **(D)** KEGG GSEA showing the top pathways negatively enriched in the *EIF2S1*-high group. **(E)** GO over-representation analysis of FDR-significant DEGs. **(F)** KEGG over-representation analysis of FDR-significant DEGs. Adjusted *p* values were calculated using the Benjamini-Hochberg method.

Transcriptomic profiling comparing *EIF2S1*-high and *EIF2S1*-low AML cohorts identified a total of 373 DEGs, including 276 upregulated and 97 downregulated genes, using limma with empirical Bayes moderation and the predefined thresholds of |log_2_ fold change| > 1 and adjusted *p* < 0.05 (**Figure 3A**). Ranked-list gene set enrichment analysis (GSEA) showed positive enrichment of immune-related programs in the *EIF2S1*-high group, including antigen processing and presentation, MHC class II protein complex assembly, and cellular defense responses. Additional inflammation- and immune-response-related pathways were also enriched (**Figures 3B, C**). Conversely, negatively enriched pathways predominantly involved metabolic and regulatory processes, including ascorbate and aldarate metabolism and spliceosome function (**Figure 3D**). GO analysis of the DEG set identified enrichment in inflammatory-response regulation, leukocyte migration, responses to bacterial-origin molecules and lipopolysaccharide, membrane- and secretory-granule-associated cellular components, and immune- and pattern-recognition-receptor activities (**Figure 3E**). KEGG analysis further identified enrichment in phagocytosis, neutrophil extracellular trap formation, neuroactive ligand-receptor interaction, and selected metabolic and pathogen-response-labeled pathways (**Figure 3F**). The pathogen-response-labeled pathways were interpreted as reflecting shared innate immune, inflammatory, and phagocytic gene modules rather than evidence of specific infectious processes. Collectively, these findings associate *EIF2S1*-high AML with antigen-presentation-, inflammatory-, innate immune-, leukocyte-migration-, and phagocytosis-related transcriptional programs, together with alterations in selected metabolic and RNA-processing pathways.

### 3.4 *EIF2S1* expression is associated with myeloid- and T-cell-related transcriptional features in AML

**Figure 4:**
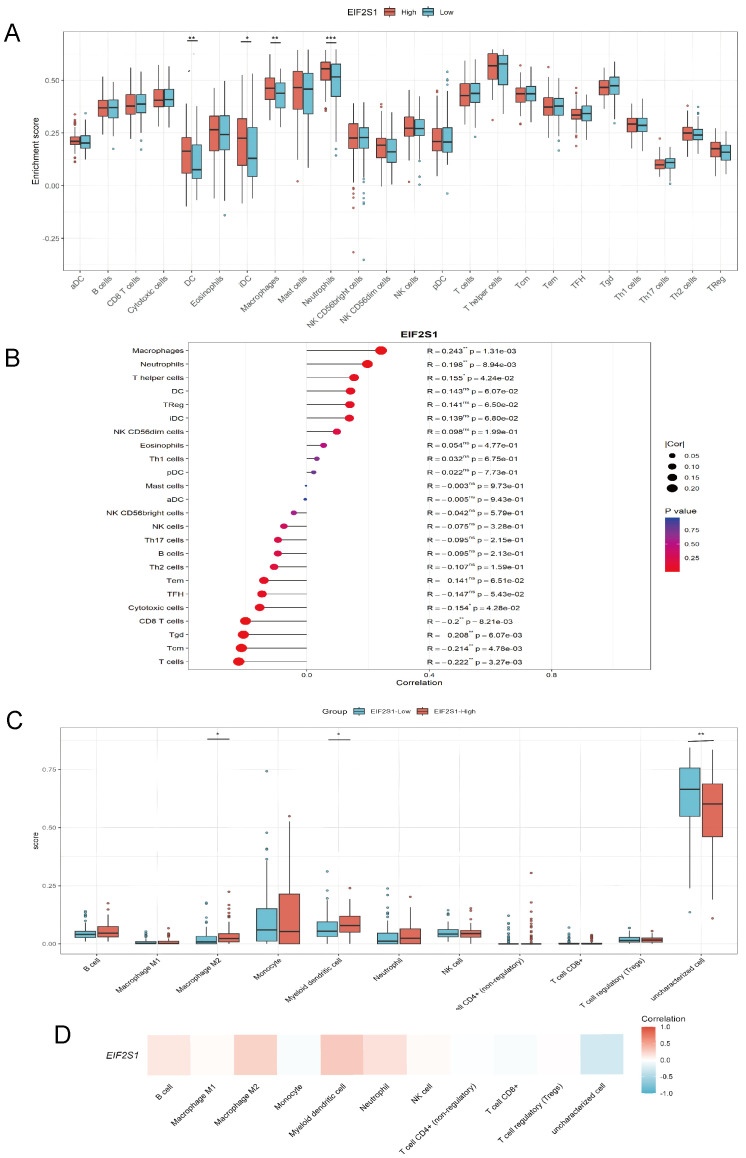
Immune-cell-related transcriptional features associated with *EIF2S1* expression in AML. **(A)** Relative enrichment scores for predefined immune-cell-associated gene signatures calculated using ssGSEA in the *EIF2S1*-high (n = 86) and *EIF2S1*-low (n = 87) groups. **(B)** Spearman correlations between *EIF2S1* expression and ssGSEA-derived immune-cell signature scores. **(C)** Relative immune-cell proportions computationally estimated using quanTIseq in the *EIF2S1*-high and *EIF2S1*-low groups. **(D)** Spearman correlations between continuous *EIF2S1* expression and quanTIseq-estimated cell proportions. Group comparisons were performed using the two-sided Mann-Whitney U test. *p* values were adjusted separately across ssGSEA-derived signatures and quanTIseq-estimated cell types using the Benjamini-Hochberg method. ssGSEA scores reflect the relative enrichment of cell-type-associated transcriptional signatures, whereas quanTIseq values represent computationally estimated cell proportions.

To characterize immune-cell-related transcriptional features associated with *EIF2S1* expression in AML, we applied two complementary computational approaches, ssGSEA and quanTIseq. ssGSEA analysis showed that continuous *EIF2S1* expression was positively correlated with macrophage- and neutrophil-related signature scores, whereas inverse correlations were observed with total T-cell-, CD8-positive T-cell-, and cytotoxic-cell-related signature scores (**Figures 4A, B**). Complementary quanTIseq analysis estimated higher relative proportions of M2-like macrophages and myeloid dendritic cells in the *EIF2S1*-high group (**Figures 4C, D**). Because these analyses were based on bulk RNA-sequencing data, the findings were interpreted as immune- and myeloid-lineage-related transcriptional features rather than direct evidence of altered immune-cell infiltration. To examine the potential influence of monocytic AML composition, we performed sensitivity analyses according to FAB subtype. *EIF2S1* expression did not differ significantly between FAB M4/M5 and non-M4/M5 cases. After exclusion of FAB M4/M5 cases, the positive association between *EIF2S1* and the macrophage-related ssGSEA score remained evident. Several additional myeloid- and T-cell-related associations remained directionally consistent. The quanTIseq analysis also continued to show positive correlation patterns between *EIF2S1* and selected myeloid-cell estimates, including M2-like macrophages and myeloid dendritic cells (**Supplementary Figure 5**).

### 3.5 *EIF2S1* correlates with immune checkpoint-related genes and heterogeneous checkpoint expression patterns

**Figure 5:**
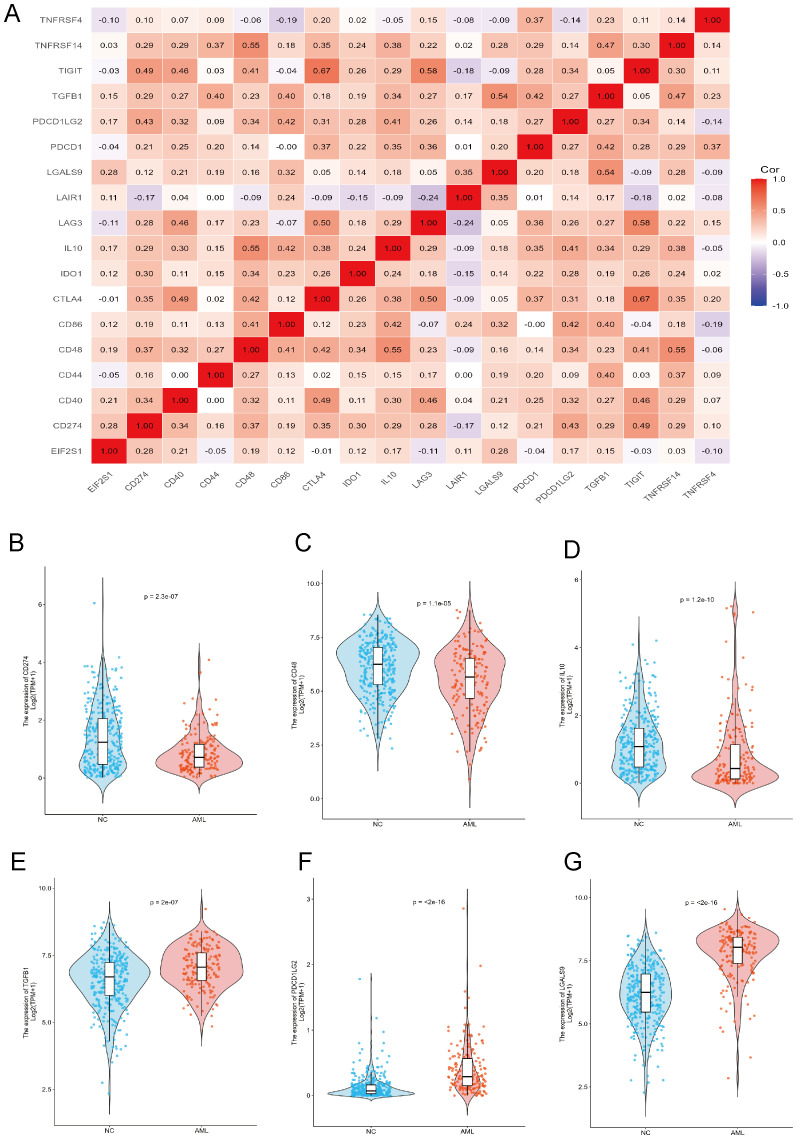
Correlation between *EIF2S1* and immune checkpoint gene expression. **(A)** Heatmap showing the correlation coefficients between *EIF2S1* and a panel of 19 immune checkpoint genes. **(B–G)** Comparison of mRNA expression levels of key immune checkpoint genes (B: *CD274*, C: *CD48*, D: *IL10*, E: *TGFB1*, F: *PDCD1LG2*, G: *LGALS9*) between normal and AML samples. Spearman correlation analysis was performed to assess co-expression relationships between genes.

In the TCGA-LAML cohort, *EIF2S1* expression was significantly positively correlated with multiple immune checkpoint-related genes, including *CD274, CD40, CD48, IL10, LGALS9, PDCD1LG2* and *TGFB1* (**Figure 5A**; all *p* < 0.05), suggesting a potential role for *EIF2S1* in modulating the immune microenvironment in AML. Notably, differential expression analysis revealed marked heterogeneity in the expression patterns of these immune checkpoint genes in AML. Specifically, *CD274, CD48* and *IL10* were overall downregulated, whereas *LGALS9, PDCD1LG2* and *TGFB1* were significantly upregulated (**Figures 5B–G**; *p* < 0.0001). These findings indicate that the relationship between *EIF2S1* and immune checkpoint regulation may be influenced by distinct molecular backgrounds and regulatory mechanisms within AML.

### 3.6 Single-cell transcriptomics localizes *EIF2S1* enrichment to monocyte-like subsets in AML

**Figure 6:**
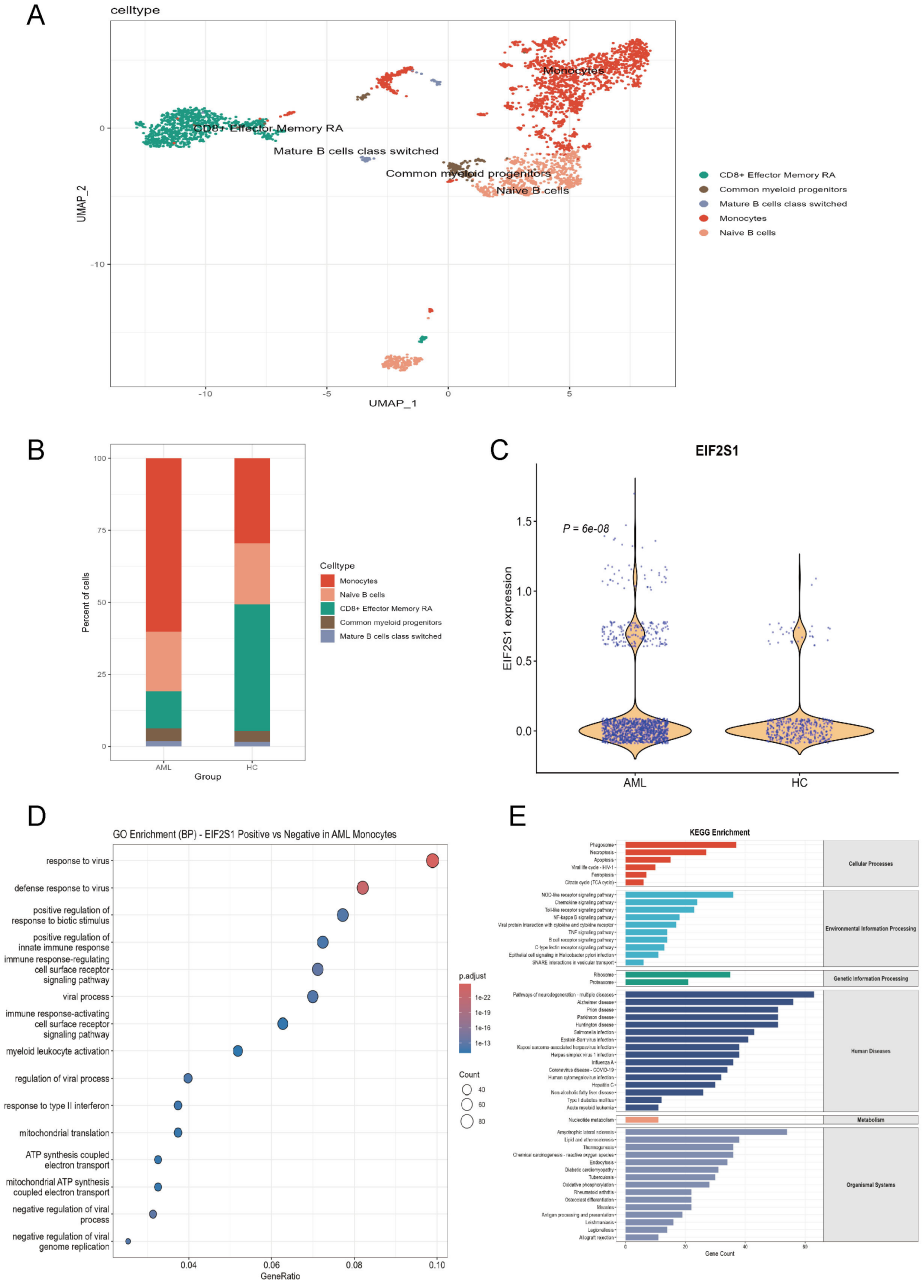
Single-cell transcriptomic analysis of *EIF2S1* in AML microenvironment. **(A)** UMAP plot showing annotations of the five major cell types in the GSE154109 dataset from the GEO database. **(B)** Bar plot comparing the proportion of each cell type in AML patients (n = 8) and healthy controls (n = 4). **(C)** Differential expression of *EIF2S1* in monocytes of AML patients and healthy controls. **(D)** Significantly enriched GO entries related to biological processes. **(E)** KEGG pathway enrichment analysis based on single-cell data. Significantly enriched KEGG pathways for differentially expressed genes (DEGs) between *EIF2S1*-positive and *EIF2S1*-negative monocyte subsets. Statistical significance of the enrichment results was defined as *q* < 0.05.

Single-cell transcriptome mapping showed that, compared with healthy controls (HC), the immune microenvironment components of AML patients underwent significant remodeling, mainly manifested as an increase in the proportion of monocytes and naive B cells, while a sharp decrease in CD8^+^ effector memory T cells (**Figures 6A, B**). Gene expression analysis showed that the expression level of the translation initiation factor *EIF2S1* was significantly upregulated in the AML microenvironment (**Figure 6C**). To explore its potential mechanism, we enriched the co-expression network of *EIF2S1* in AML monocytes. GO and KEGG analyses revealed that *EIF2S1* expression is closely related to the activation of innate immune/stress responses (such as antiviral defense responses) and mitochondrial metabolism (mitochondrial translation, ATP synthesis coupled with electron transport) (**Figures 6D, E**). In summary, AML samples exhibited marked cellular composition remodeling. *EIF2S1* enrichment in monocyte-like subsets was associated with mitochondrial metabolic programs and innate immune/stress-response signatures.

### 3.7 *EIF2S1* knockdown induces G0/G1-phase accumulation and impairs leukemic cell fitness in THP-1 cells

**Figure 7:**
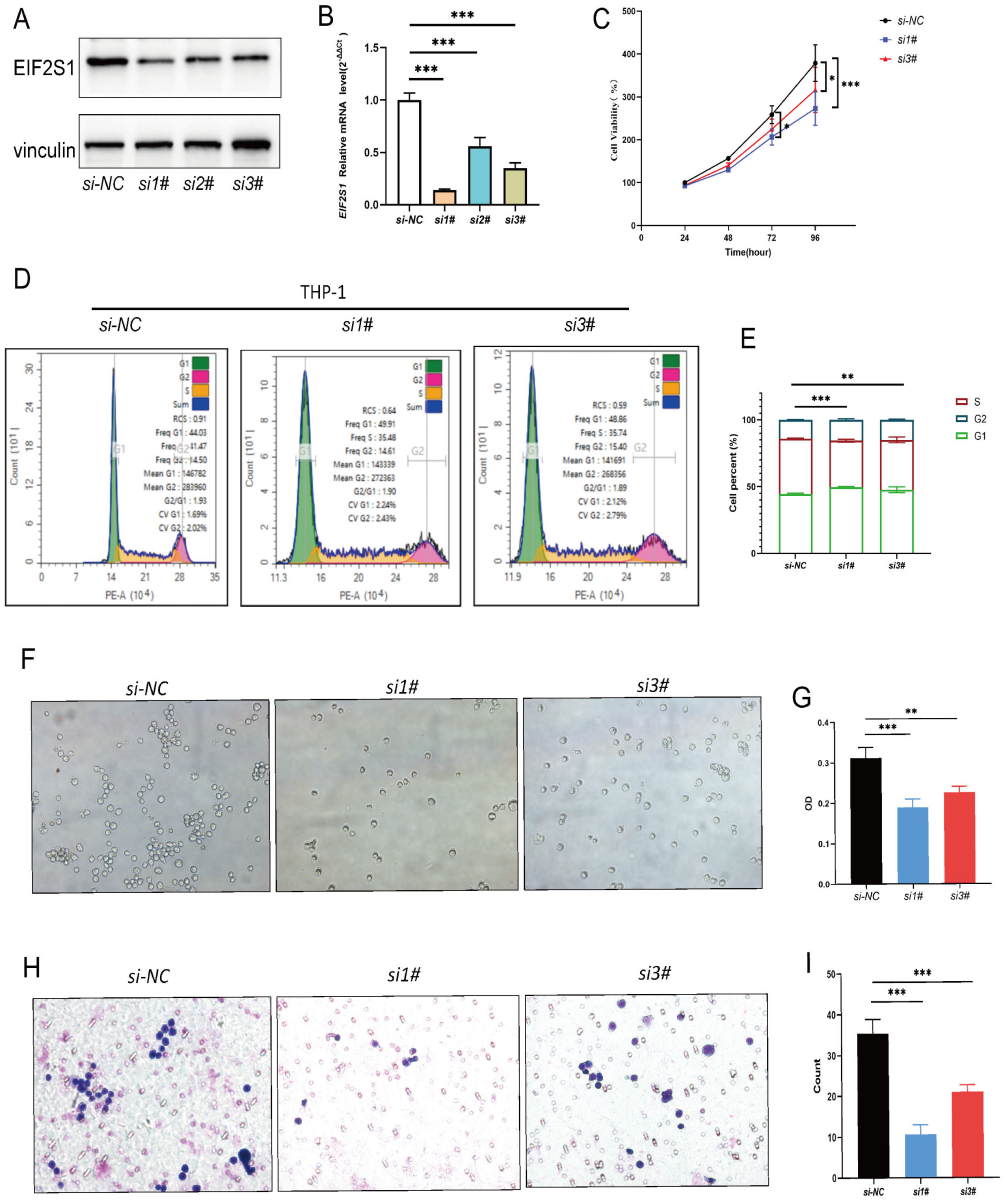
*EIF2S1* knockdown induces G0/G1-phase accumulation and impairs leukemic cell fitness in THP-1 cells. **(A)** Validation of *EIF2S1* protein knockdown in THP-1 cells. **(B)** Validation of *EIF2S1* mRNA knockdown in THP-1 cells. **(C)** Cell proliferation curves detected by CCK-8 assay. **(D, E)** Cell cycle detection by flow cytometry. **(F, G)** Transwell assay to detect cell migration ability in each group, scale bar = 50μm. **(H, I)** Transwell assay to detect cell invasion ability in each group, scale bar = 50μm. Data are expressed as mean ± SD of three independent experiments, n = 3. *p < 0.05, **p < 0.01, ***p < 0.001.

Given the specific enrichment of *EIF2S1* in monocytic subsets identified in our scRNA-seq analysis, we selected THP-1, a representative human acute monocytic leukemia cell line, to further functionally validate. We specifically knocked down *EIF2S1* in THP-1 cells using small interfering RNA (siRNA). Western blotting and RT-qPCR analysis confirmed that *si1#* and *si3#* were the most efficient and were used in subsequent experiments (**Figures 7A, B**). Cell kinetic analysis showed that the loss of *EIF2S1* significantly inhibited the proliferation rate of THP-1 cells (**Figure 7C**). Flow cytometry further showed that knockdown of *EIF2S1* led to significant G0/G1 phase cell cycle arrest (**Figures 7D, E**). Furthermore, *in vitro* experiments confirmed that downregulation of *EIF2S1* severely impaired the cell’s motility potential: compared with *si-NC*, the loss of *EIF2S1* greatly inhibited the migration ability of THP-1 cells (**Figures 7F, G**), and the number of invading cells penetrating the basement membrane was also significantly reduced (**Figures 7H, I**). *EIF2S1* knockdown increased the proportion of apoptotic cells in THP-1 cultures (**Supplementary Figure 6**; **Supplementary Table 1**). Collectively, these findings indicate that *EIF2S1* knockdown suppresses proliferation, induces G0/G1-phase accumulation, promotes apoptosis, and impairs migration and invasion in THP-1 cells.

## 4 Discussion

While the application of novel targeted therapies has made AML treatment more personalized, chemotherapy resistance and relapse remain major clinical challenges (28, 29). Immunotherapy has shown great promise in solid tumors, but its efficacy in AML is heavily limited by the highly immunosuppressive bone marrow microenvironment (11, 30). Emerging evidence reveals that the establishment of this immunosuppressive niche relies heavily on aberrant protein synthesis and metabolic adaptation (31, 32). Dysregulated translational control is increasingly recognized as an important mechanism linking cancer cell adaptation, immune regulation, and therapeutic resistance (33). At the core of this process is the translation initiation factor *EIF2S1*, widely recognized as a central regulator controlling the rate-limiting step in cellular protein synthesis (34). In this study, by integrating transcriptomic data from large-scale public cohorts with single-cell RNA sequencing (scRNA-seq) and *in vitro* functional assays, we systematically delineated the expression landscape, clinical prognostic value, and biological impact of *EIF2S1* in AML. Our findings suggest that *EIF2S1* upregulation is associated with adverse prognosis, translation-initiation programs, monocyte-like immune-metabolic features, and malignant phenotypes in monocytic AML cells.

First, we corroborated the elevated expression of *EIF2S1* in AML across independent cohorts and obtained external support for its adjusted association with shorter overall survival. More importantly, multivariate Cox regression analysis revealed that high *EIF2S1* expression retains independent prognostic value even after adjusting for traditional factors such as patient age, initial cytogenetic risk, and common FLT3 and NPM1 mutations. Given that traditional genomic stratification sometimes fails to fully capture the dynamic functional status of AML at the transcriptomic and translational levels (35–37), the evaluation of *EIF2S1* could serve as a valuable supplement to existing molecular markers, enabling more precise identification of high-risk patients in clinical practice.

At the molecular level, our interaction network and co-expression analyses placed *EIF2S1* within a tightly coordinated translation initiation-associated module in AML. *EIF2S1* expression was positively correlated with key components of the eIF2 and eIF3 machinery, including EIF2B3, EIF2S3, and EIF3B, indicating coordinated expression of translation-initiation-related genes in *EIF2S1*-high AML. Such a transcriptional pattern may reflect the increased biosynthetic demands of rapidly proliferating leukemic cells, which require sustained protein synthesis to support cell-cycle progression and malignant growth. This interpretation is consistent with our functional findings that *EIF2S1* knockdown induced G0/G1 cell-cycle arrest, suppressed THP-1 cell proliferation and promoted apoptosis.

Beyond maintaining autonomous cell survival, the crosstalk between leukemia cells and the bone marrow immune microenvironment dictates disease progression (38, 39). Analysis using limma with empirical Bayes moderation and FDR-controlled thresholds yielded a more conservative DEG set, while the principal enrichment patterns remained broadly consistent. Ranked-list GSEA associated *EIF2S1*-high AML with antigen-processing, inflammatory, and immune-response programs, whereas negatively enriched pathways included selected metabolic and RNA-processing processes. GO and KEGG analyses further highlighted inflammatory-response regulation, leukocyte migration, innate immune recognition, phagocytosis, and membrane- or secretory-granule-associated functions. Several KEGG categories carried infectious-disease labels; however, these pathways likely reflect shared innate immune, inflammatory, and phagocytic gene modules rather than evidence of specific infectious processes. Crucially, *EIF2S1* expression exhibited a positive correlation with multiple immune checkpoints (such as *LGALS9, TGFB1*, and *PDCD1LG2*). It is biologically plausible that the synthesis of certain immunomodulatory factors harboring complex mRNA structures heavily depends on the overactivation of translation initiation pathways (40). Consistently, ssGSEA and quanTIseq analyses associated higher *EIF2S1* expression with stronger myeloid-lineage-related transcriptional signals, including higher macrophage-related signature scores and higher estimated relative proportions of M2-like macrophages and selected myeloid-cell populations, together with weaker cytotoxic CD8^+^T-cell-related signatures. Selected myeloid-related associations remained after exclusion of FAB M4/M5 cases, suggesting that they were not entirely attributable to monocytic AML composition. Collectively, these findings indicate that *EIF2S1*-high AML is associated with an immune- and myeloid-lineage-related transcriptional context characterized by increased checkpoint-related gene expression and weaker cytotoxic T-cell-related signatures. To resolve this complex cellular ecosystem at higher resolution, our scRNA-seq analysis characterized the microenvironmental heterogeneity. We found that *EIF2S1* was enriched in monocyte-like subsets within AML samples. Pathway analysis indicated that this high *EIF2S1* expression is tightly linked to highly active innate immune signaling networks and accelerated mitochondrial metabolic processes (including ATP synthesis and the electron transport chain). These findings suggest that *EIF2S1*-high monocyte-like subsets exhibit coordinated mitochondrial metabolic and innate immune transcriptional features at the single-cell level.

Consistent with the transcriptomic association between *EIF2S1* and monocyte-like AML states, THP-1 knockdown experiments showed that *EIF2S1* supports leukemic cell proliferation, cell-cycle progression, migration, and invasion. These findings provide functional evidence for the oncogenic relevance of *EIF2S1* in monocytic AML cells.

We acknowledge some limitations of this study. *In vitro* validation was limited to THP-1 cells, a monocytic AML model, chosen based on the association between *EIF2S1* and monocytic leukemia status in our single-cell analysis. All functional conclusions are limited to THP-1 cells. Further investigation is needed using other AML cell lines, primary patient-derived cells, and appropriate *in vivo* models, combined with other mechanistic experiments, to more comprehensively elucidate the role of *EIF2S1*. Second, although transcriptomic analyses linked *EIF2S1* to translation initiation-related programs, the study did not directly assess ribosome occupancy and translational efficiency. Further studies using ribosome profiling, polysome analysis, proteomics, and direct translation assays are needed to define *EIF2S1*-dependent translational programs. Third, the pharmacological tractability, therapeutic selectivity, and safety of *EIF2S1* targeting were not evaluated. These issues should be examined in genetically diverse AML models, primary samples, normal hematopoietic controls, and appropriate *in vivo* systems. Finally, although the adjusted prognostic association of *EIF2S1* received external support in the Beat AML 2.0 cohort, further validation in additional independent, clinically harmonized, and prospectively collected cohorts is required. Collectively, these studies will be necessary to determine whether *EIF2S1* can be developed as a safe and clinically relevant therapeutic target in AML.

In summary, *EIF2S1* is upregulated in AML and independently associated with adverse survival outcomes. Integrative bulk and single-cell transcriptomic analyses indicate that *EIF2S1*-high AML is characterized by translation initiation-related programs, monocyte-like immune-metabolic states, and immunosuppressive transcriptional features. Functional studies further support a role for *EIF2S1* in maintaining leukemic cell fitness, as its knockdown in THP-1 cells induces G0/G1 cell-cycle arrest and suppresses proliferation, migration, and invasion. Collectively, these findings identify *EIF2S1* as a clinically relevant prognostic biomarker and a potential therapeutic target requiring further experimental validation. Additional studies in genetically diverse AML models, primary patient samples, normal hematopoietic controls, and independent clinical cohorts are needed to clarify its underlying mechanisms, determine its therapeutic selectivity, and establish its translational relevance.

## Data Availability

The datasets presented in this study can be found in online repositories. The names of the repository/repositories and accession number(s) can be found in the article/**Supplementary Material**.
