# Supplementary material for: Dual relevance of EIF2S1 in acute myeloid leukemia: linking poor prognosis to immune-metabolic states and leukemic cell fitness
Source: Front Oncol. 2026 Aug 5;16:1922910. doi: 10.3389/fonc.2026.1922910 (PMC13485781; doi:10.3389/fonc.2026.1922910)
Supplement: Supplementary file 1 [file DataSheet1.pdf]

Figure.7A (n=3)

n=1

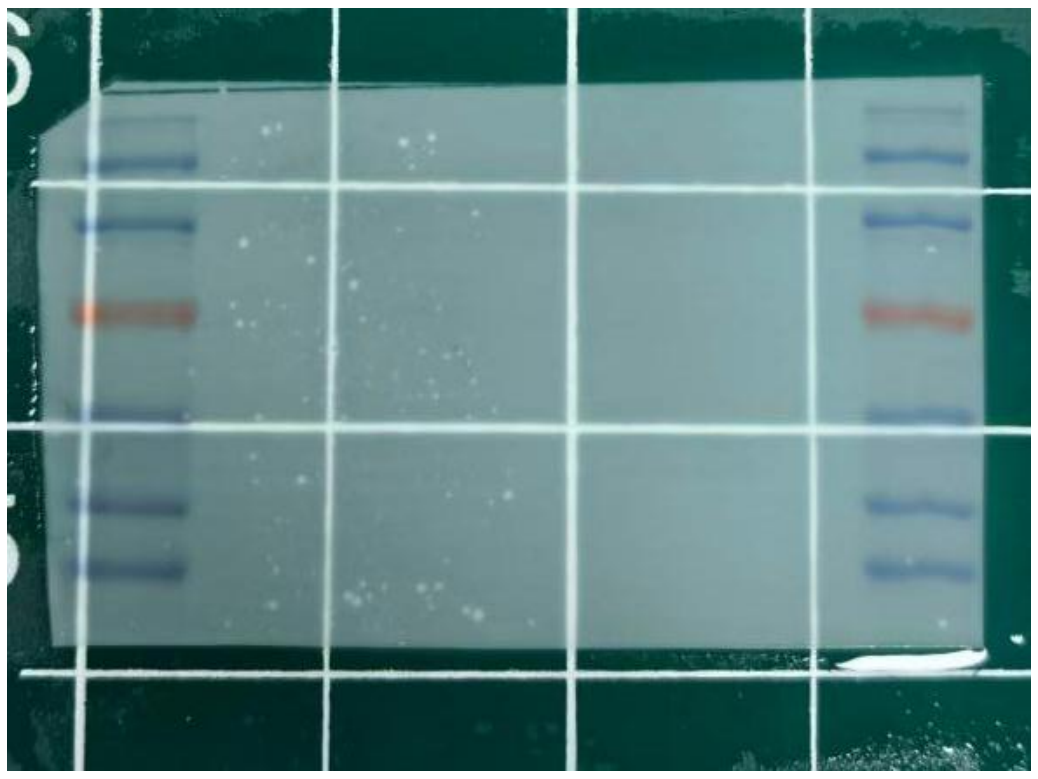

(a) The full uncropped and Blots image(s)

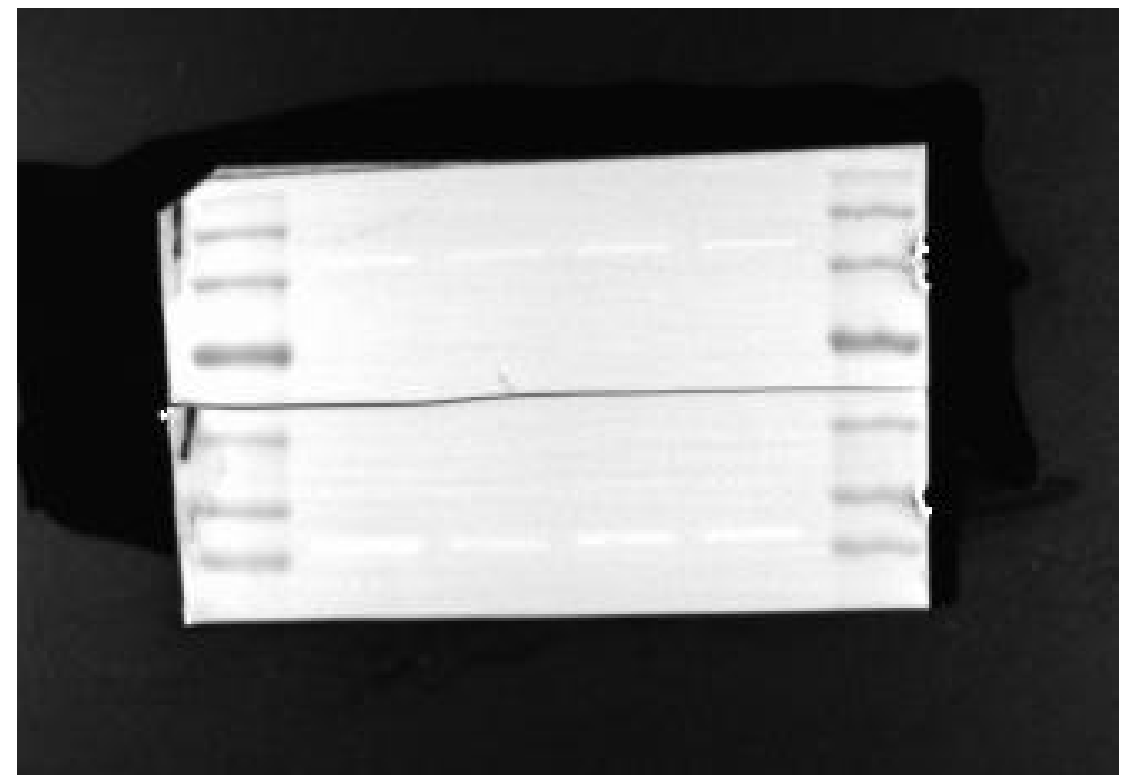

(b) Cropped and reassembled blots showing vinculin and EIF2S1

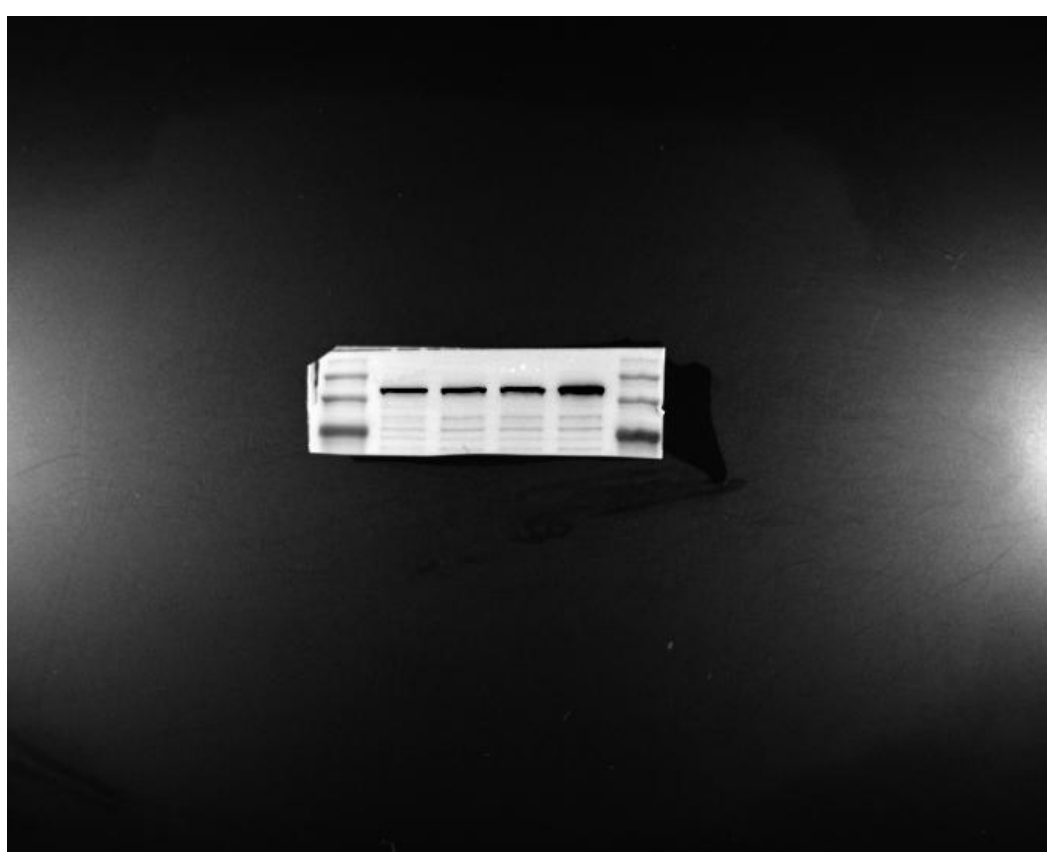

(c) Vinculin protein bands

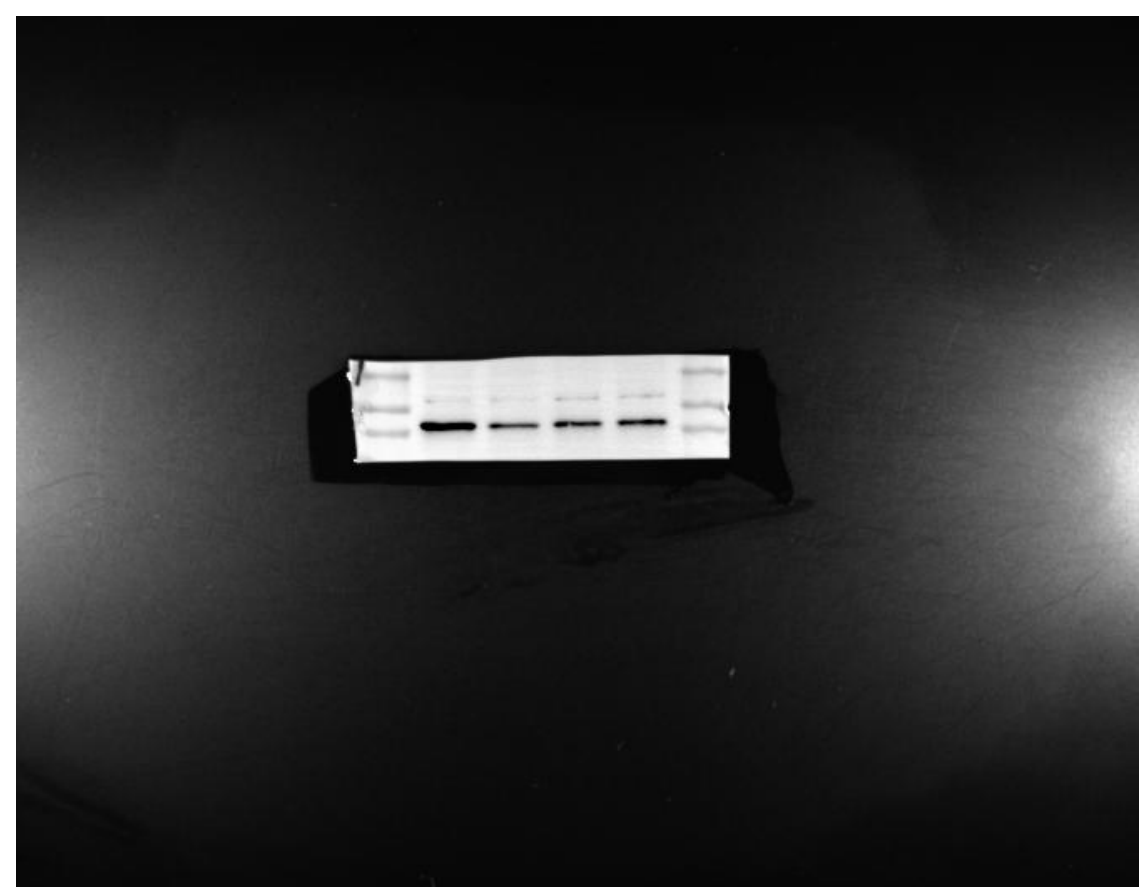

(d) EIF2S1 protein bands

n=2

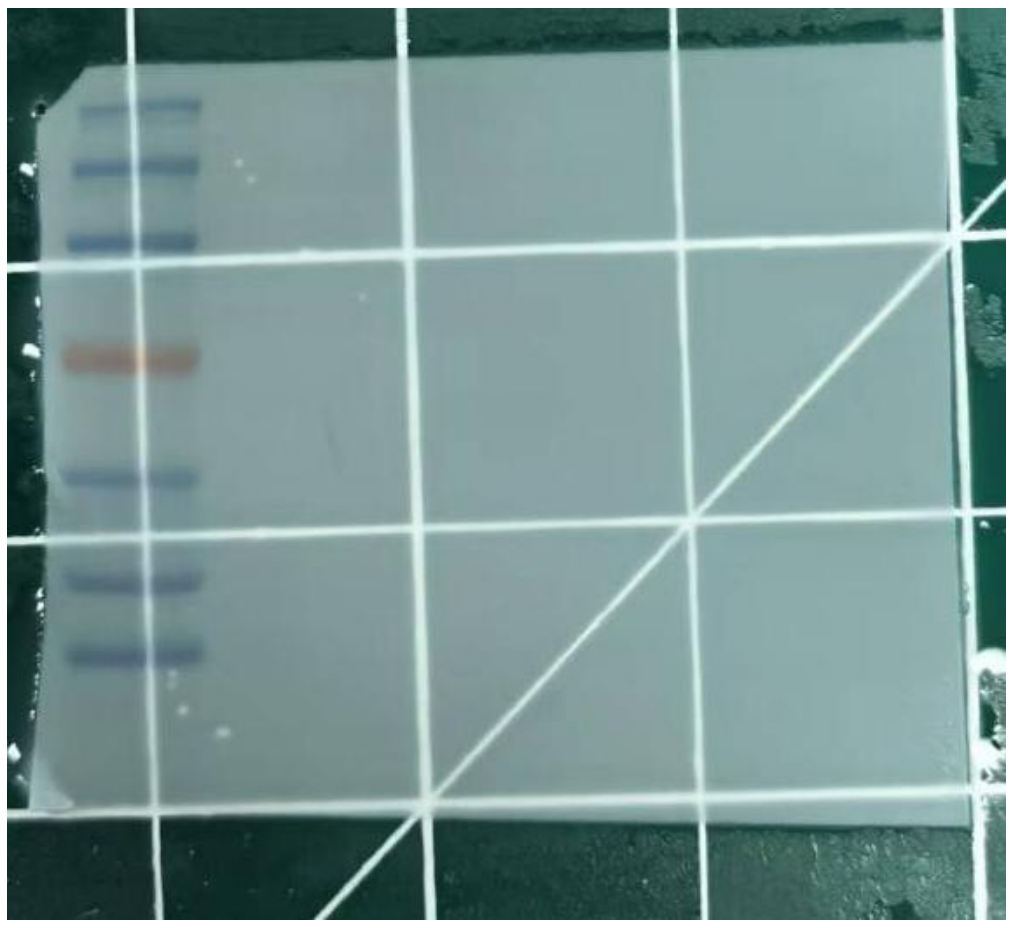

(a) The full uncropped and Blots image(s)

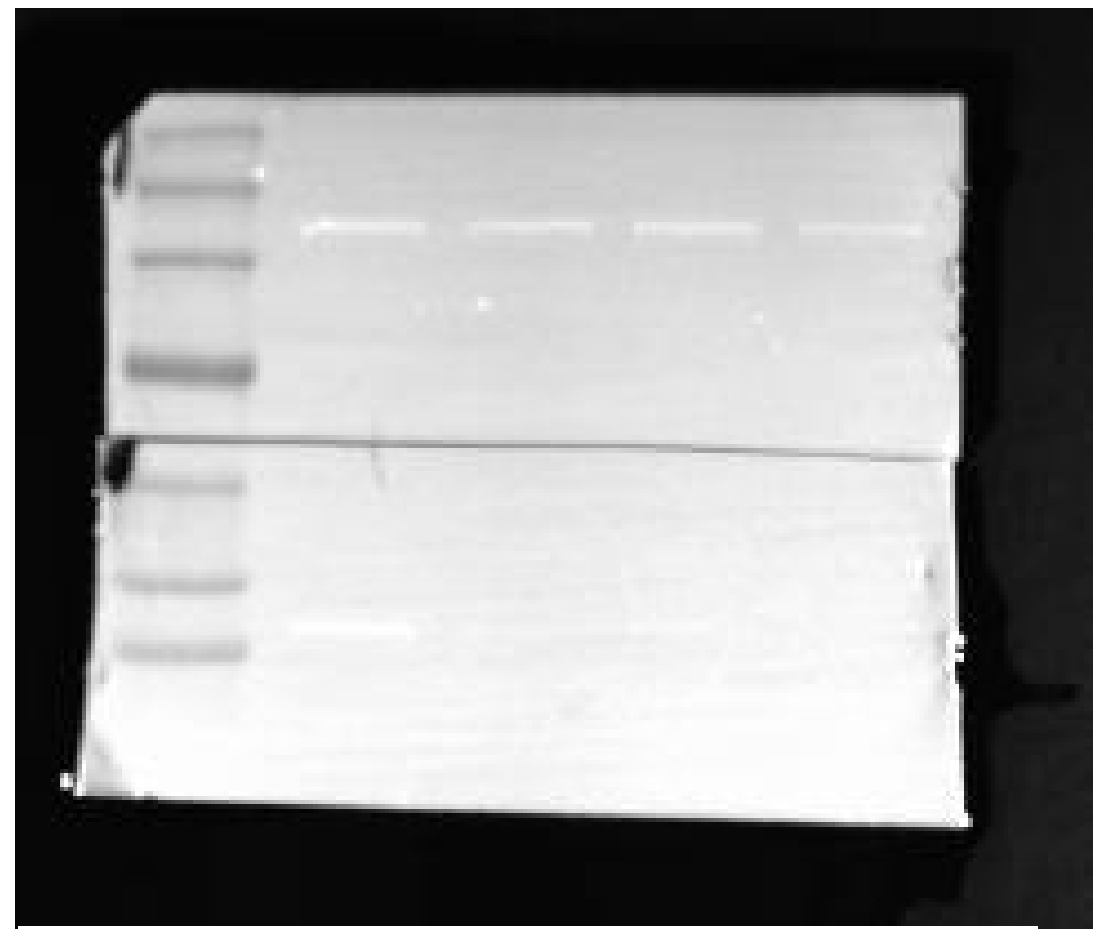

(b) Cropped and reassembled blots showing vinculin and EIF2S1

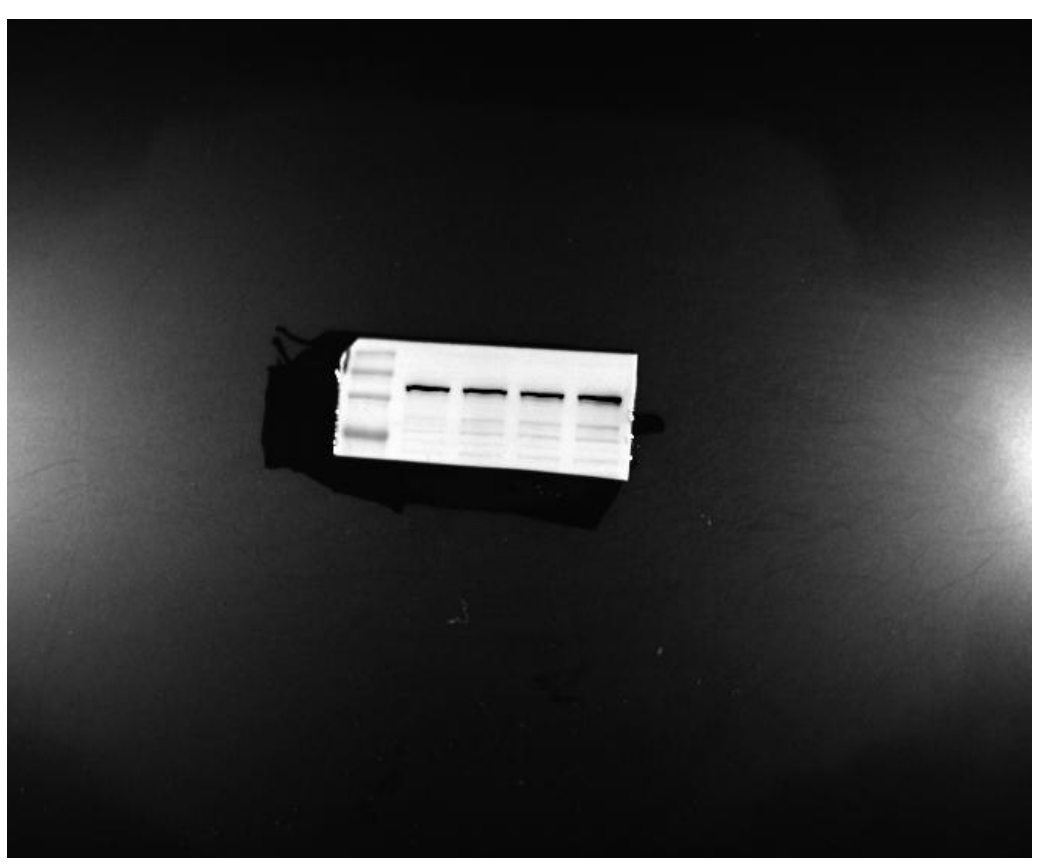

(c) Vinculin protein bands

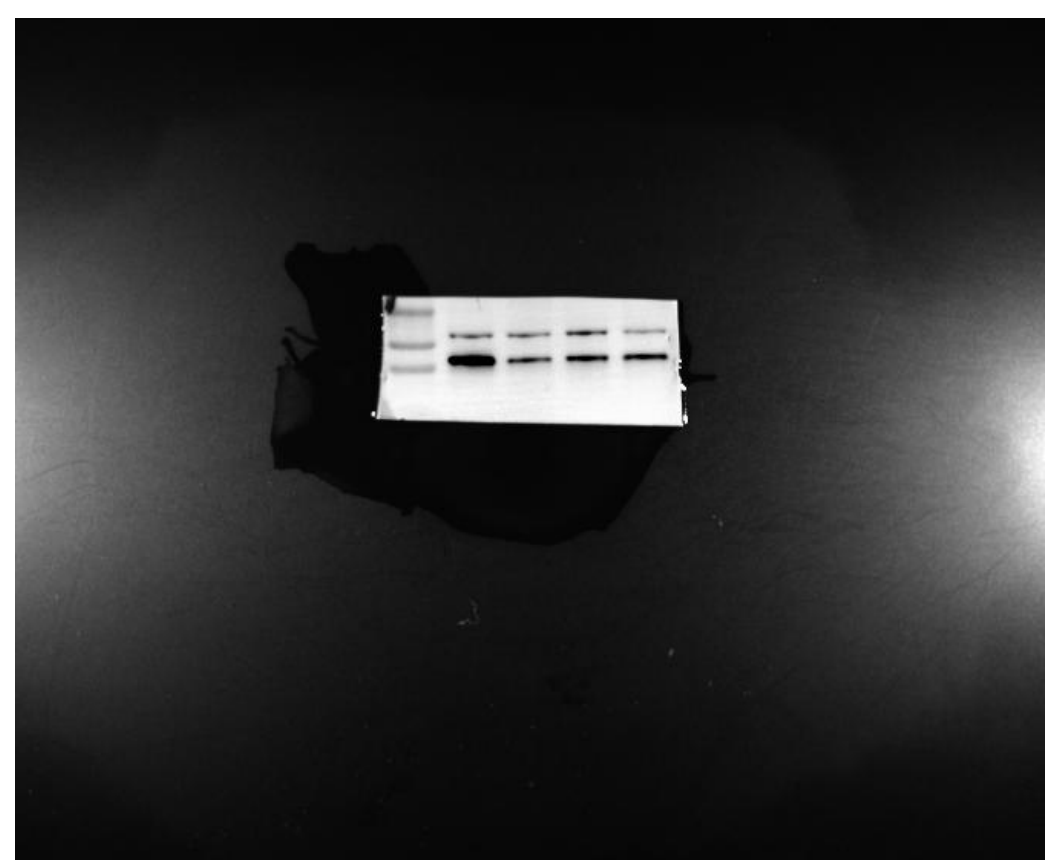

(d) EIF2S1 protein bands

n=3

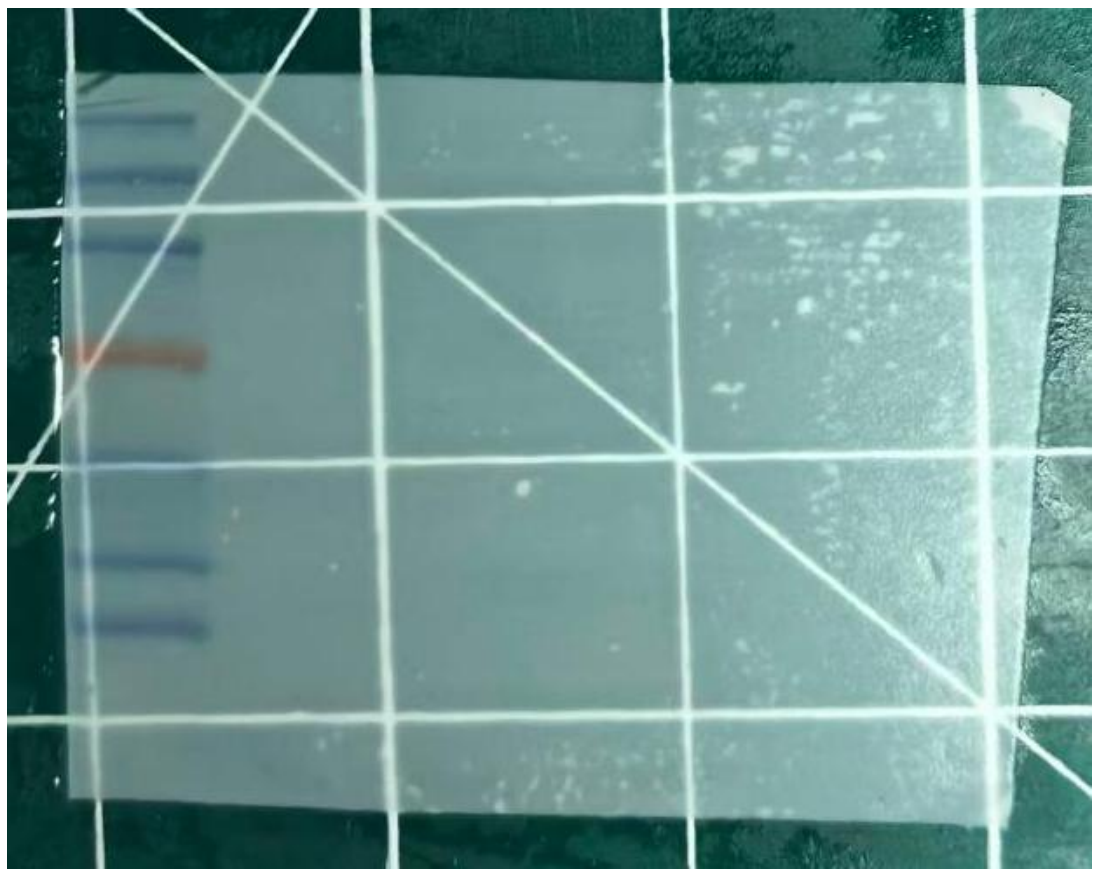

(a) The full uncropped and Blots image(s)

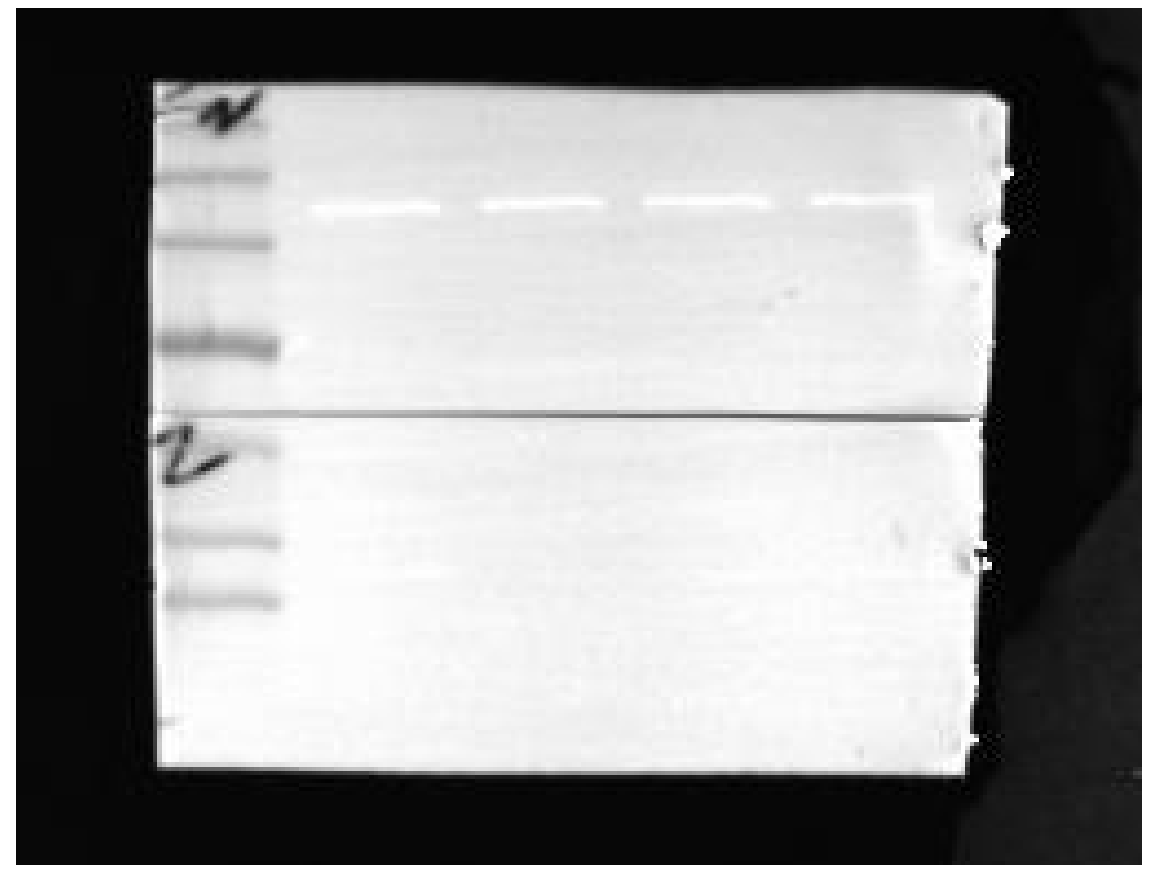

(b) Cropped and reassembled blots showing vinculin and EIF2S1

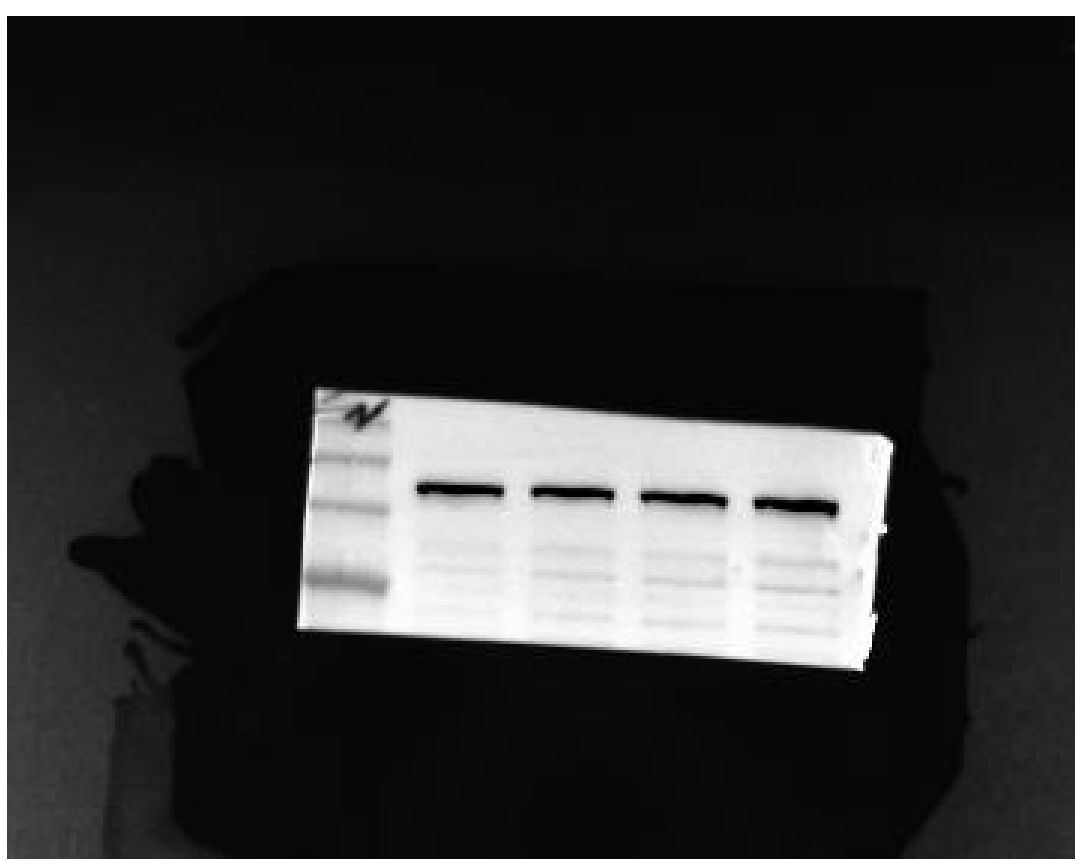

(c) Vinculin protein bands

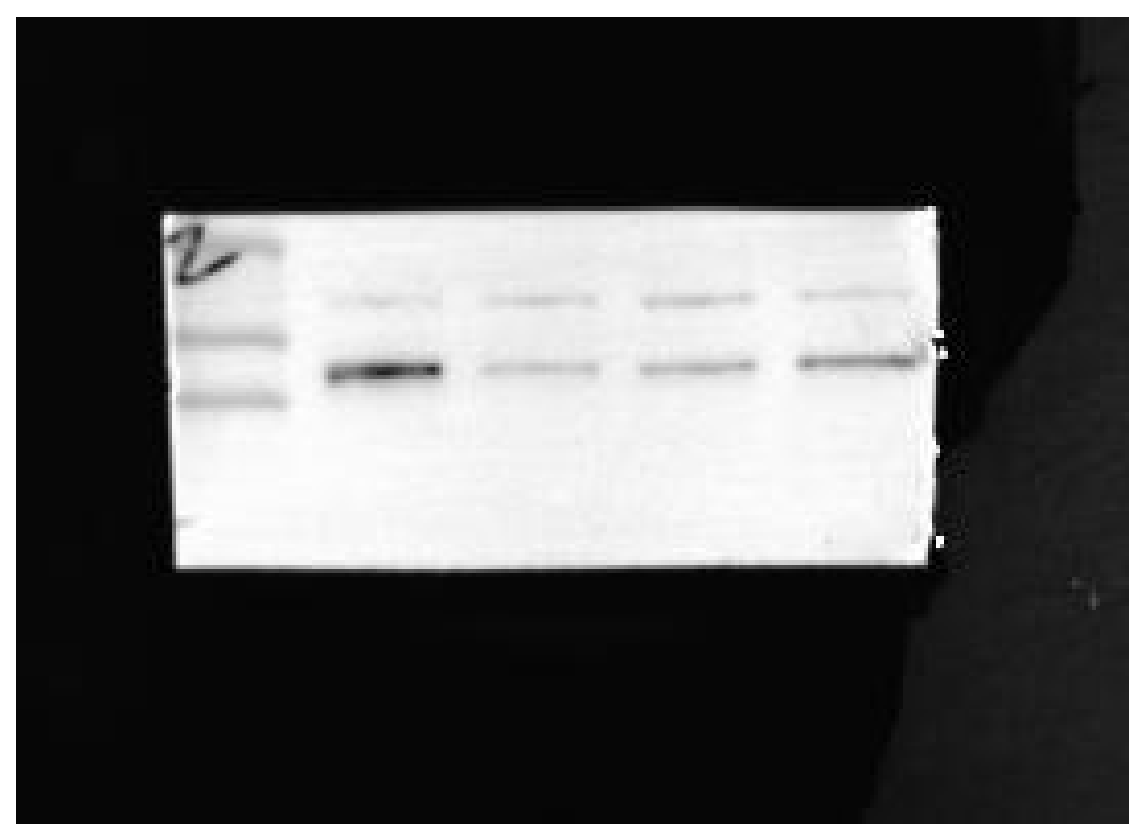

(d) EIF2S1 protein bands

Figure.7F (n=3)

1. n=1

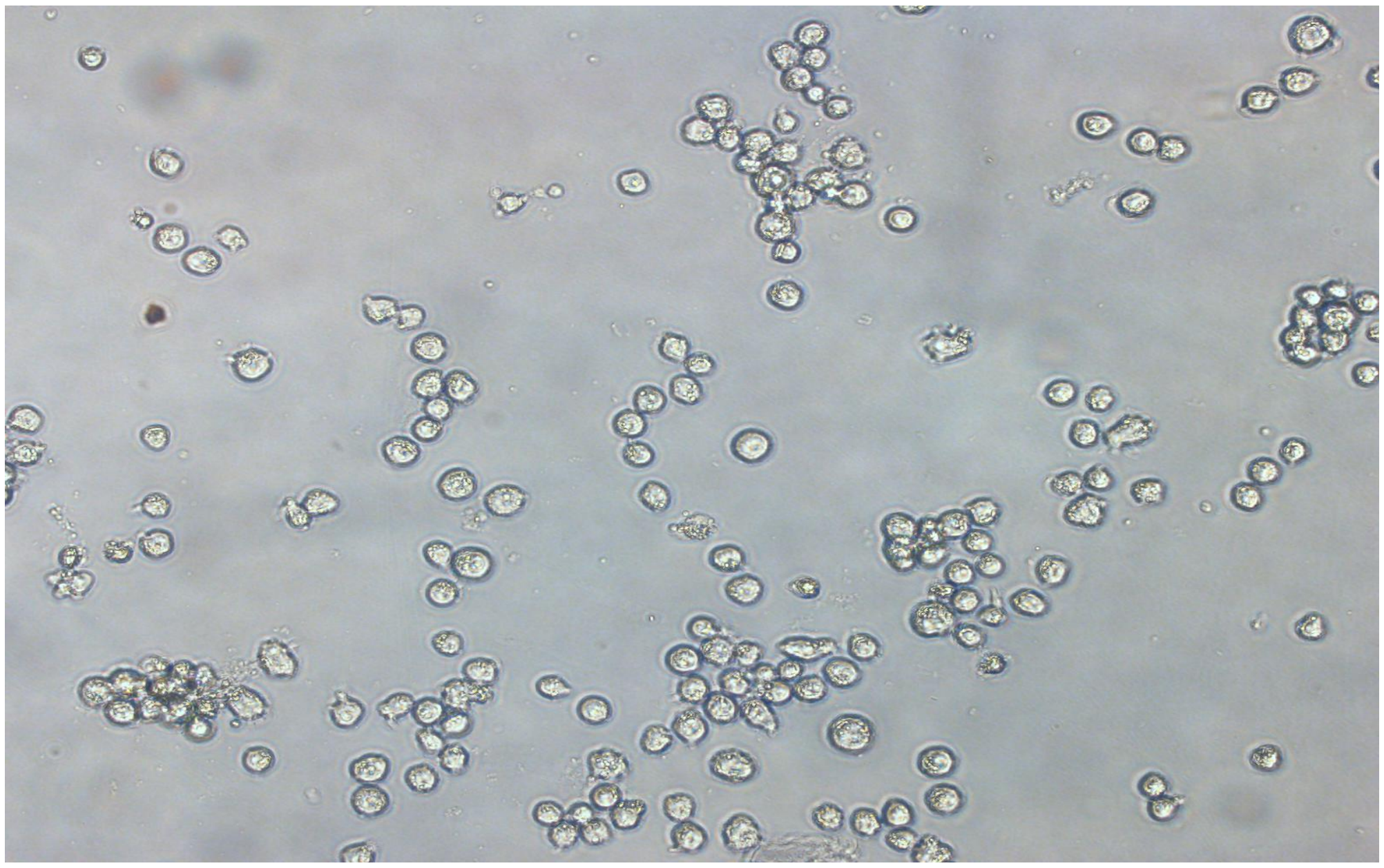

(a)*si-NC*(scale bar = 50 μm)

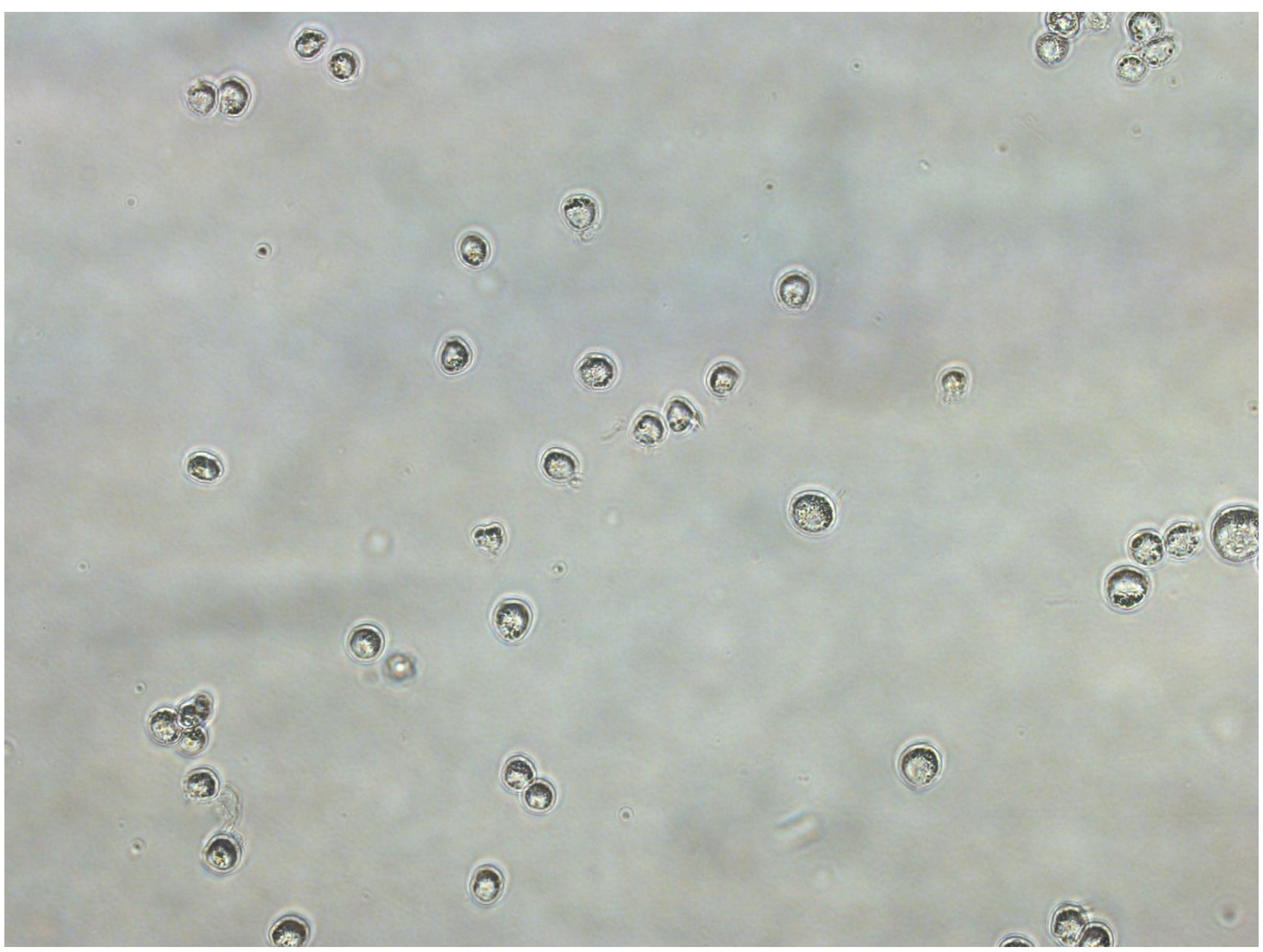

(b)*si-1#*(scale bar = 50 μm)

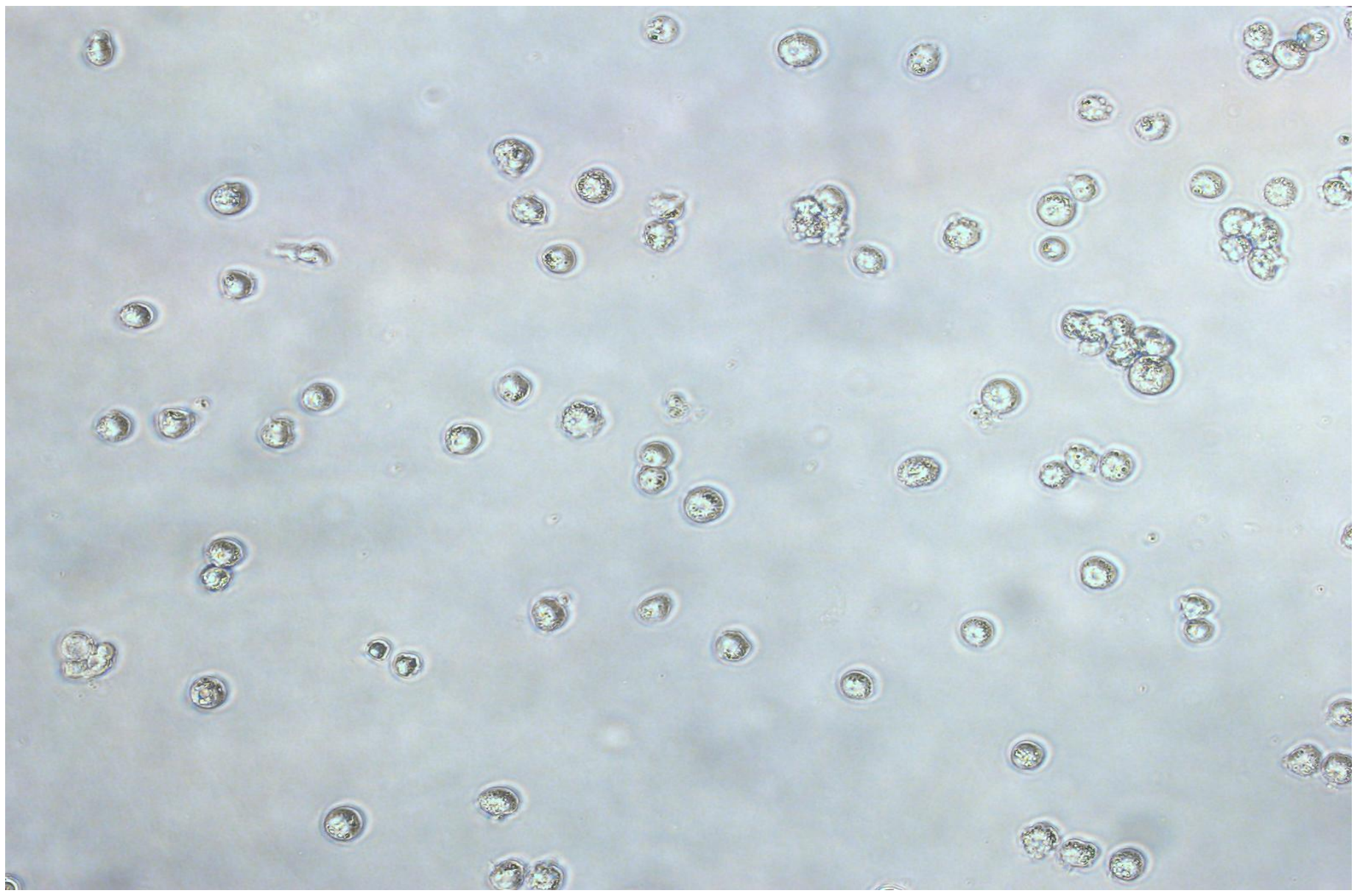

(c)*si-3#*(scale bar = 50 μm)

2. n=2

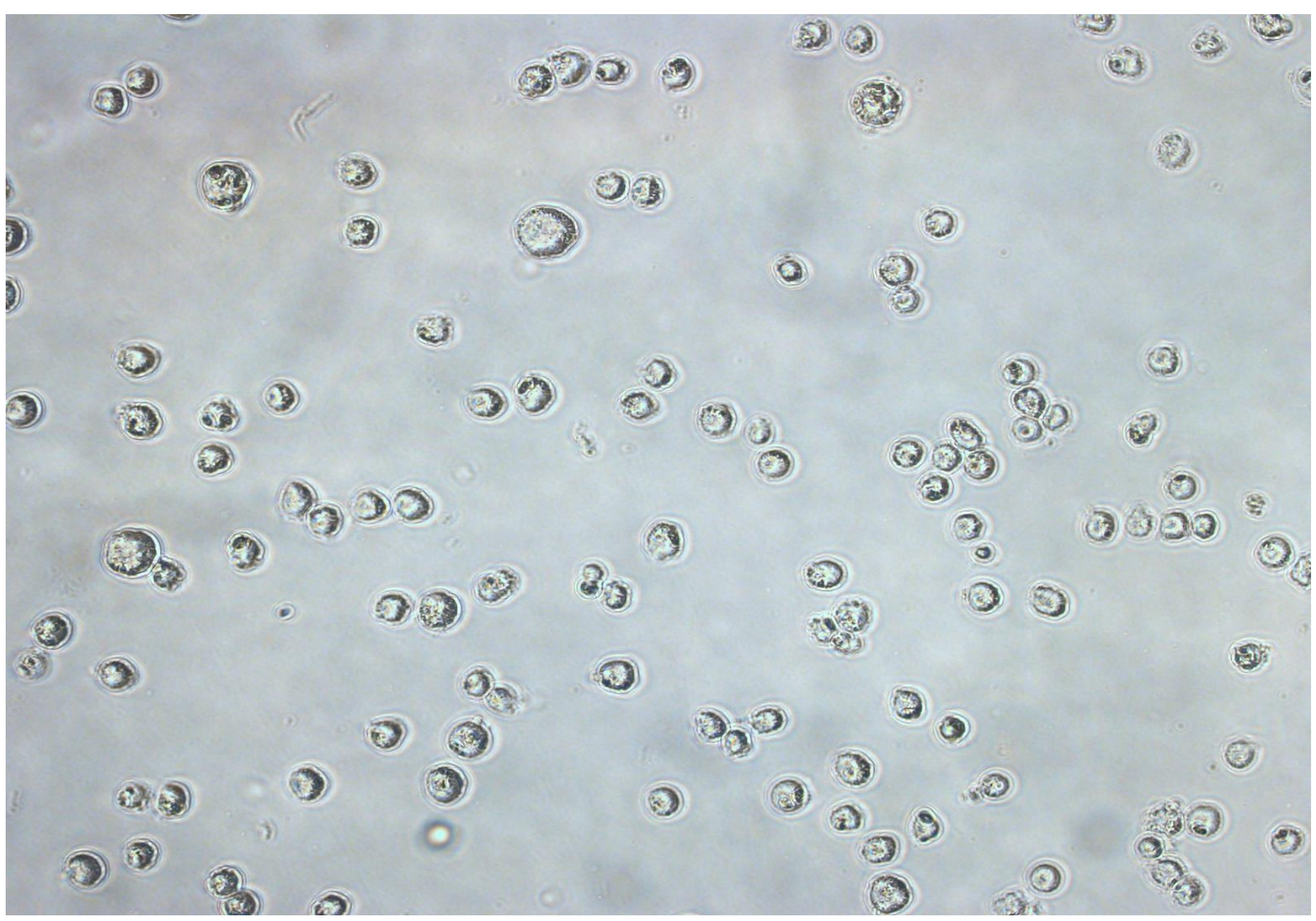

(a)*si-NC*(scale bar = 50 μm)

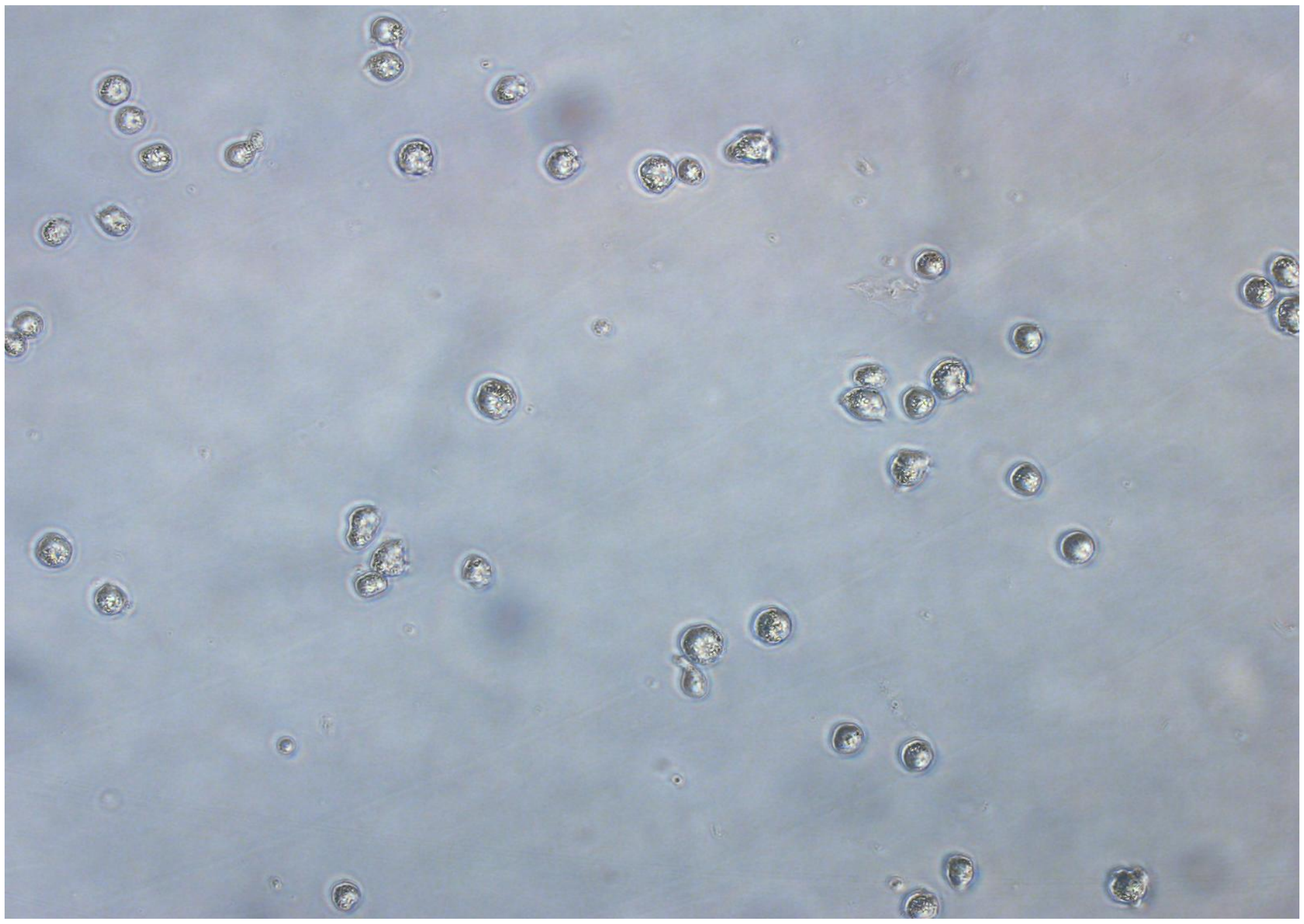

(b)*si-1#*(scale bar = 50 μm)

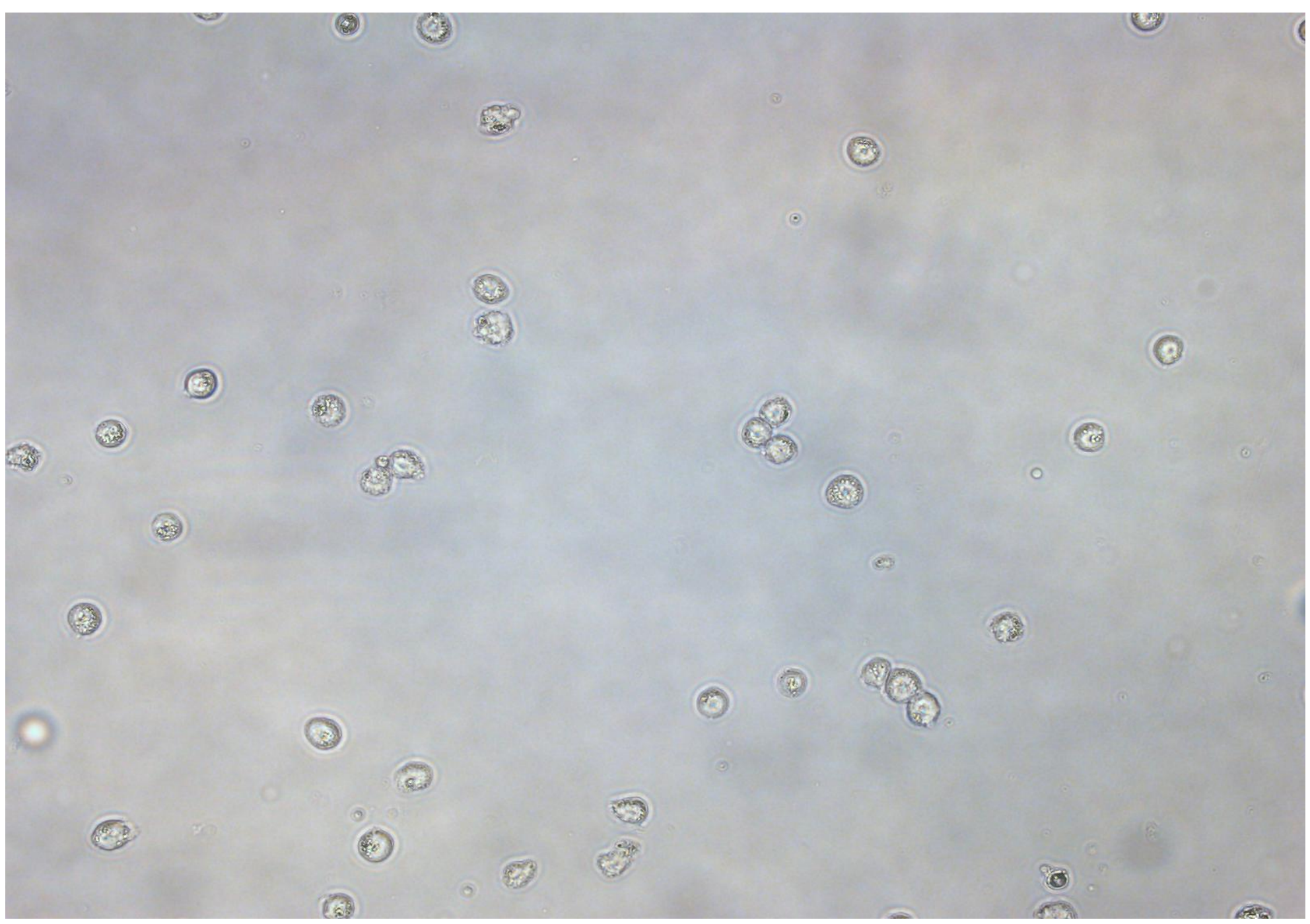

(c)*si-3#*(scale bar = 50 μm)

n=3

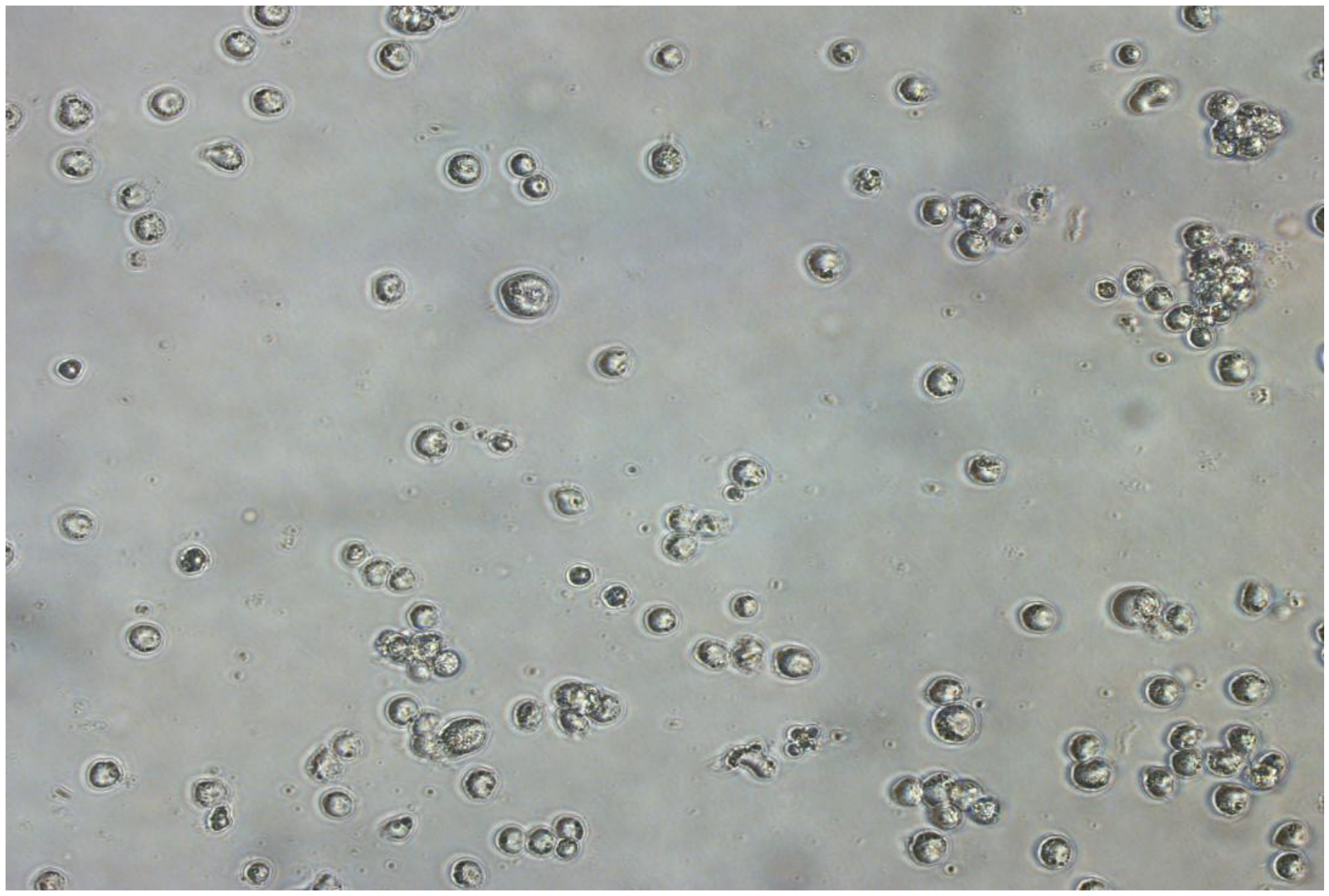

(a)*si-NC*(scale bar = 50μm)

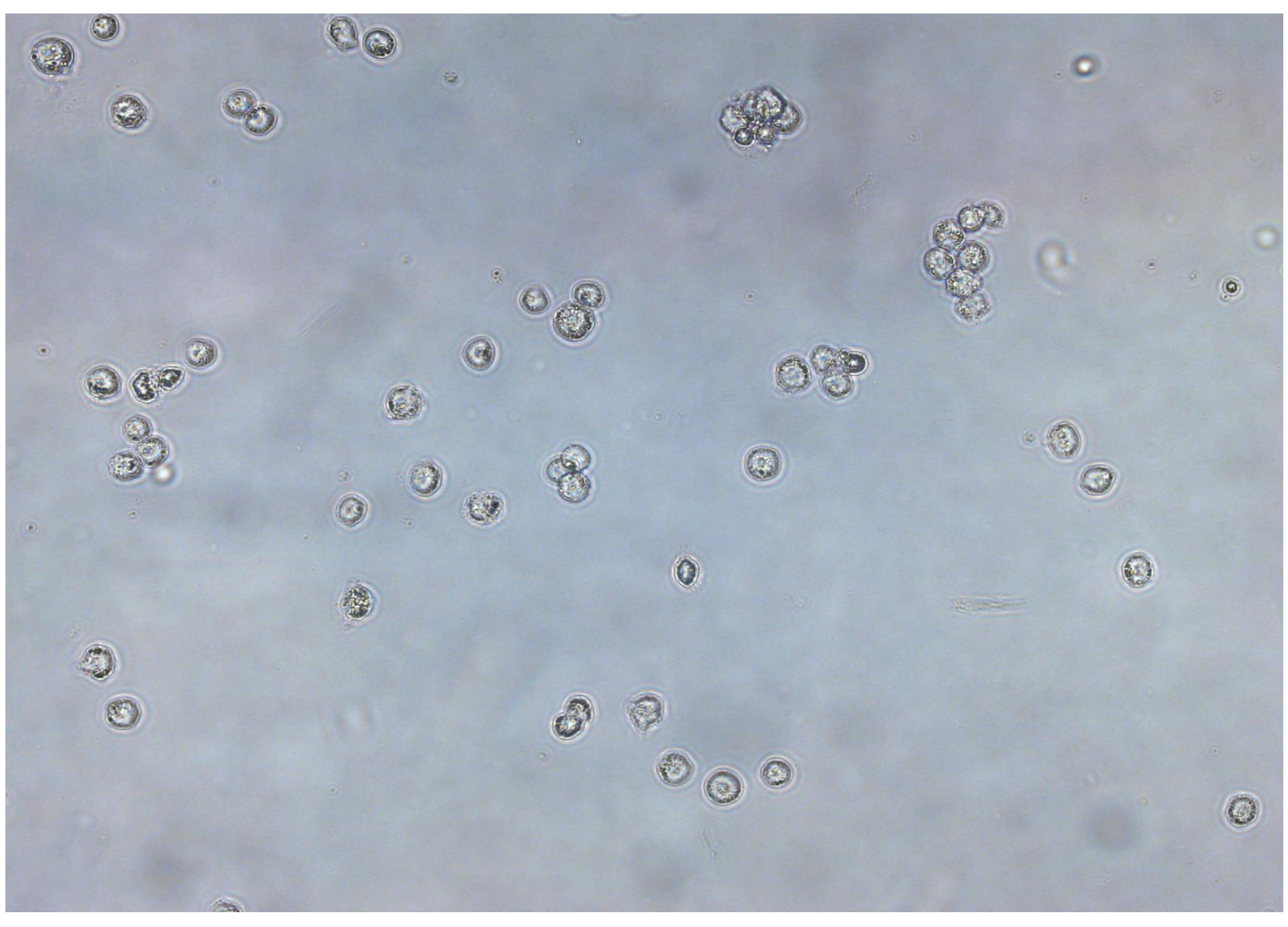

(b)*si-1#*(scale bar = 50μm)

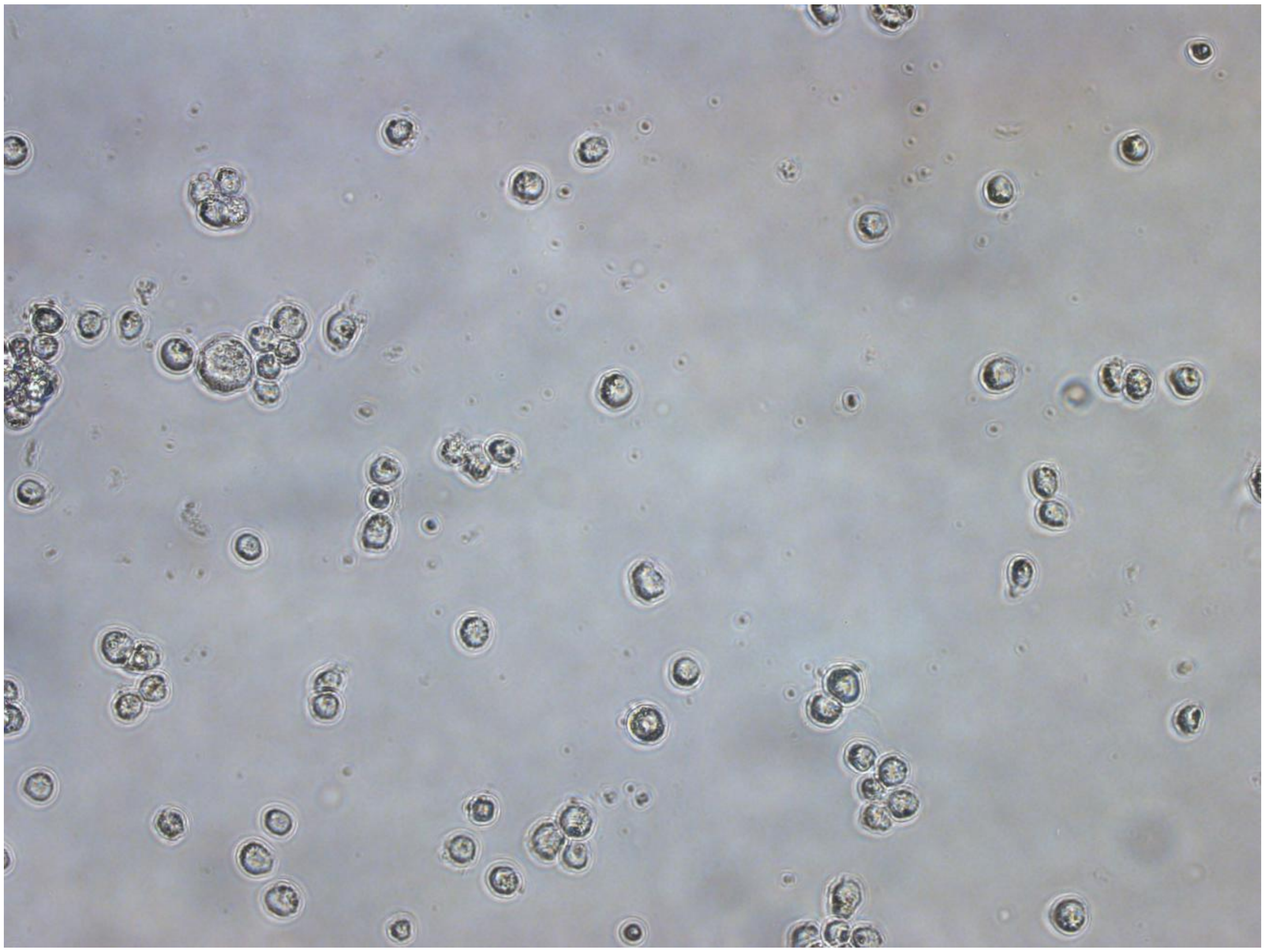

(c)*si-3#*(scale bar = 50 μm)

Figure.7H (n=3)

1. n=1

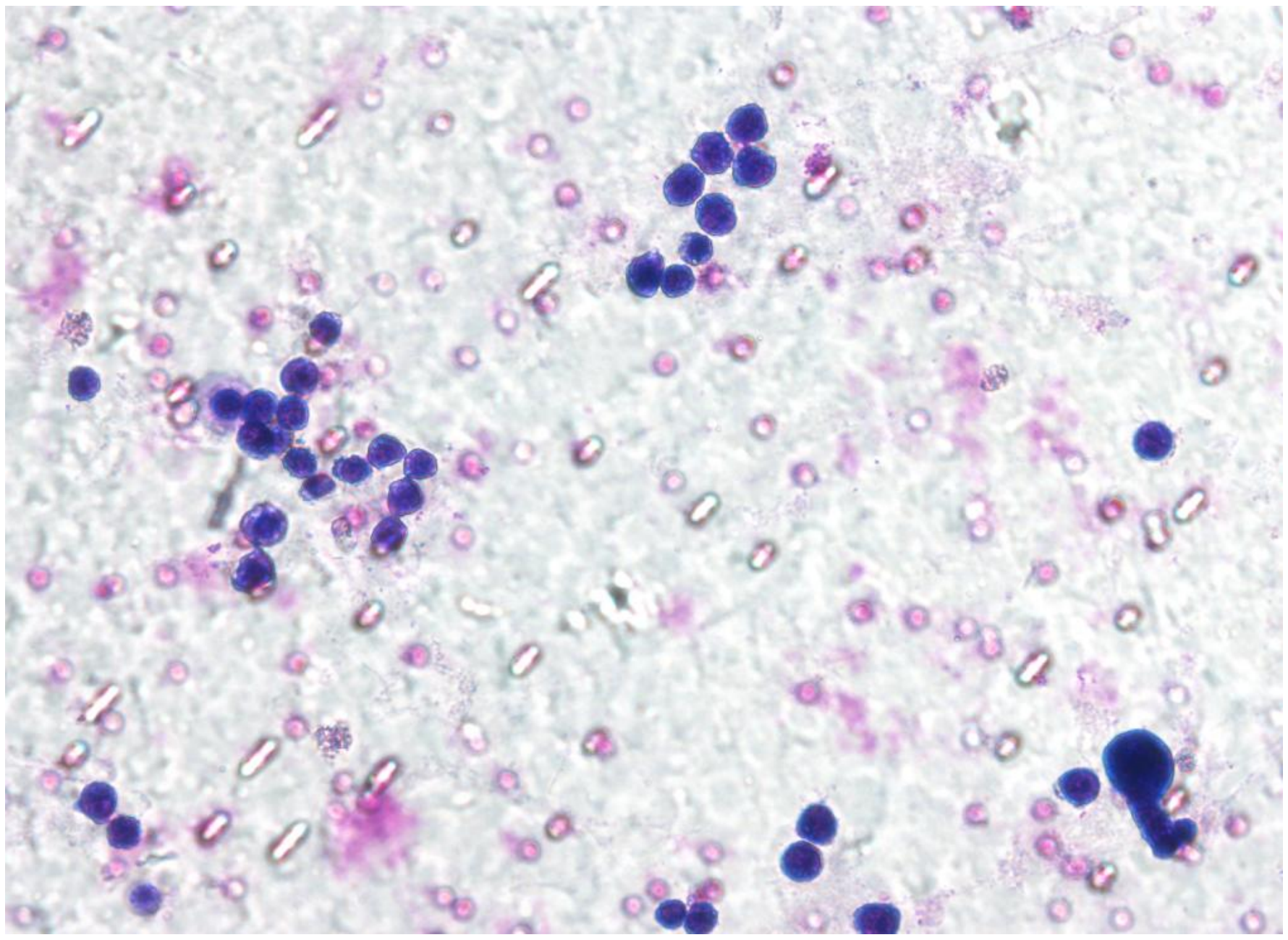

(a)*si-NC*(scale bar = 50 μm)

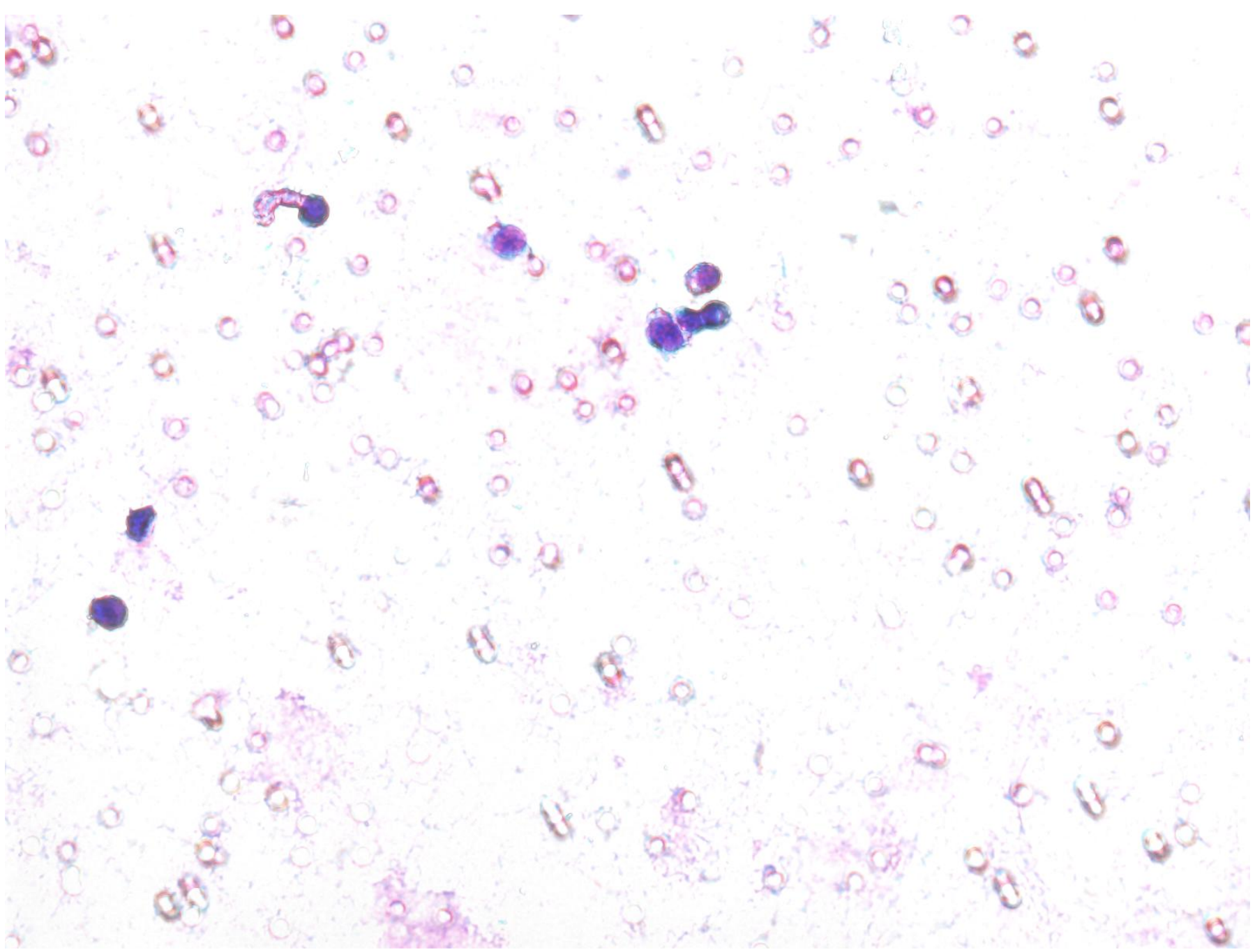

(b)*si-1#*(scale bar = 50μm)

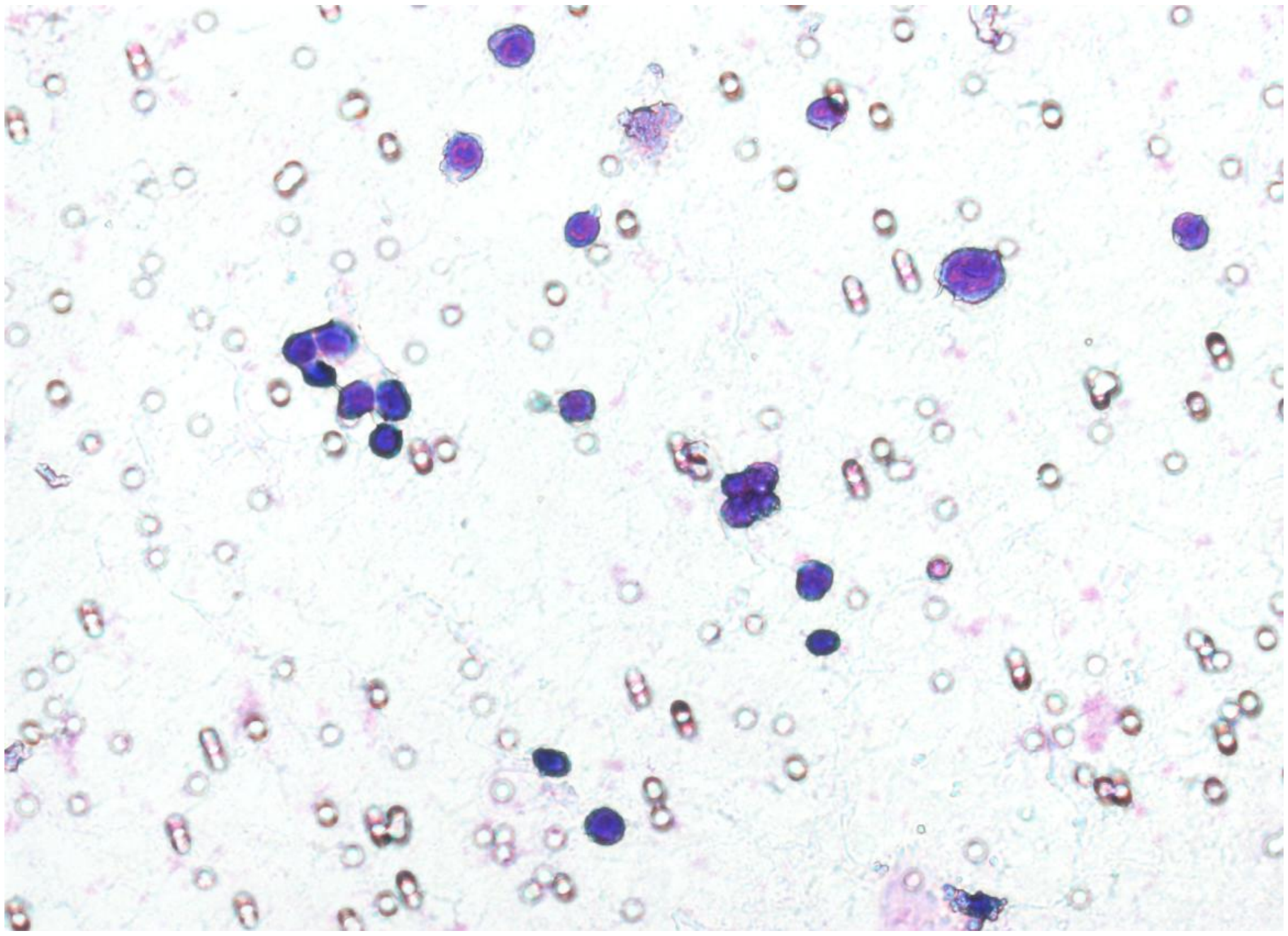

(c)*si-3#*(scale bar = 50 μm)

2. n=2

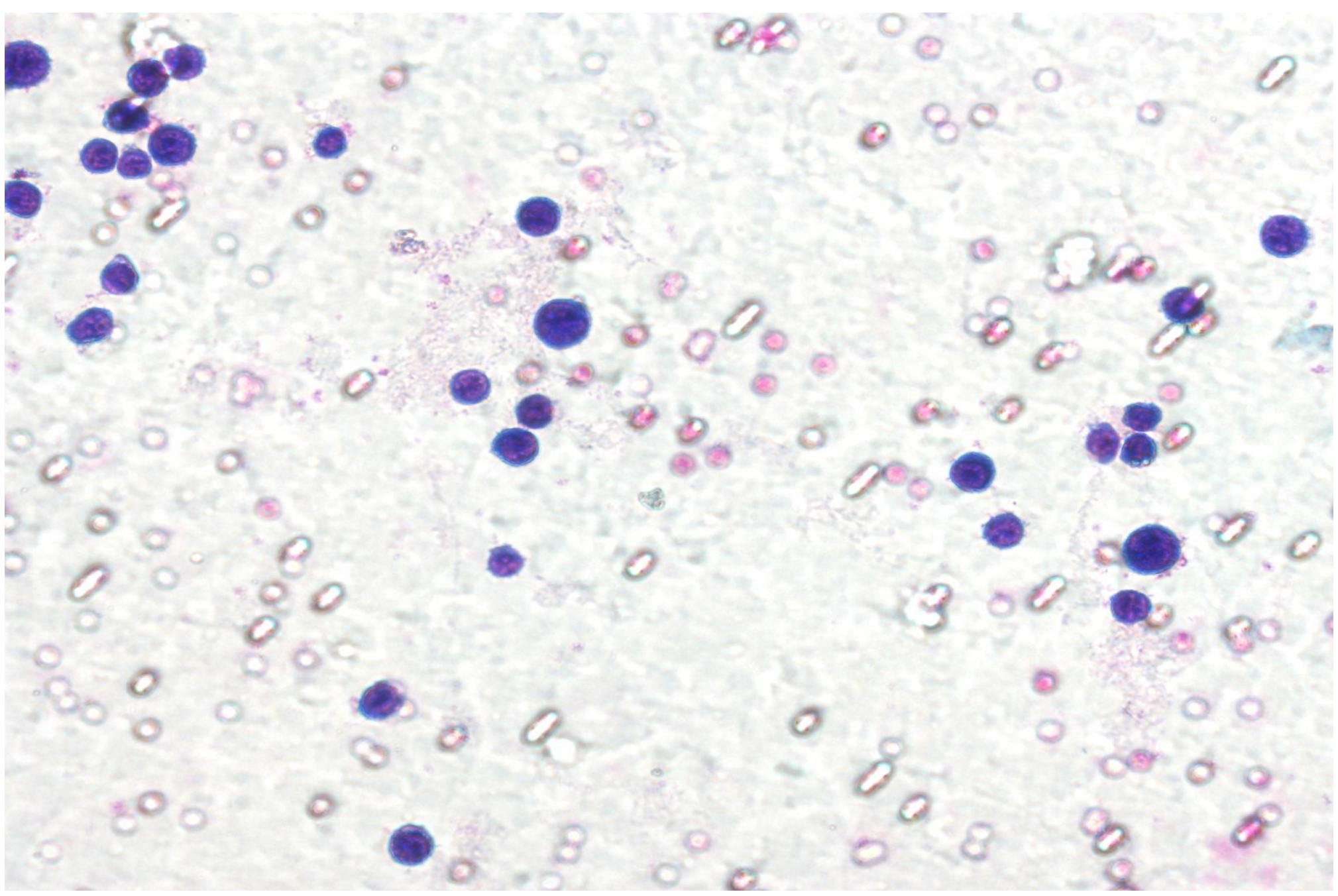

(a)*si-NC*(scale bar = 50 μm)

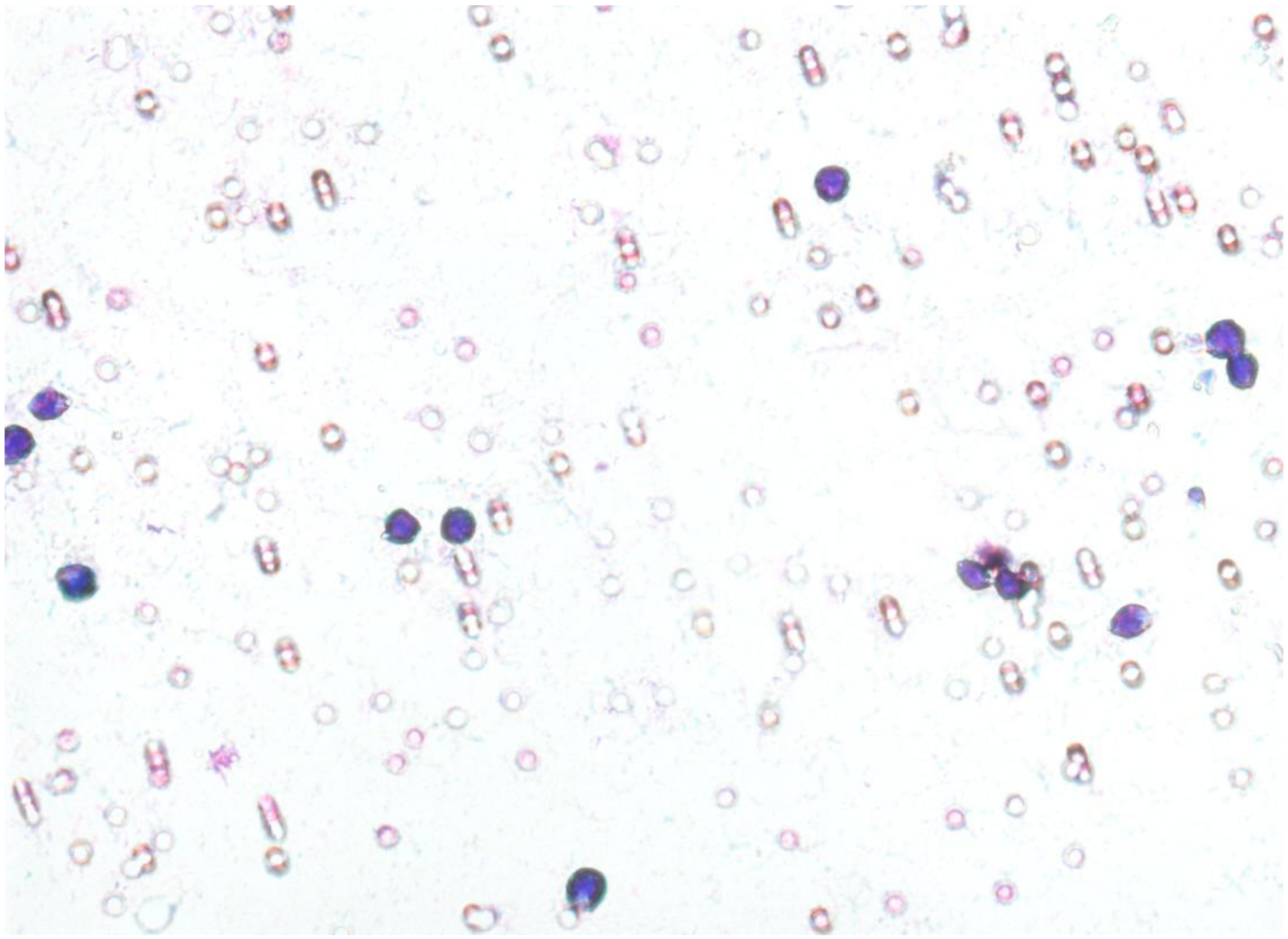

(b)*si-1*#(scale bar = 50μm)

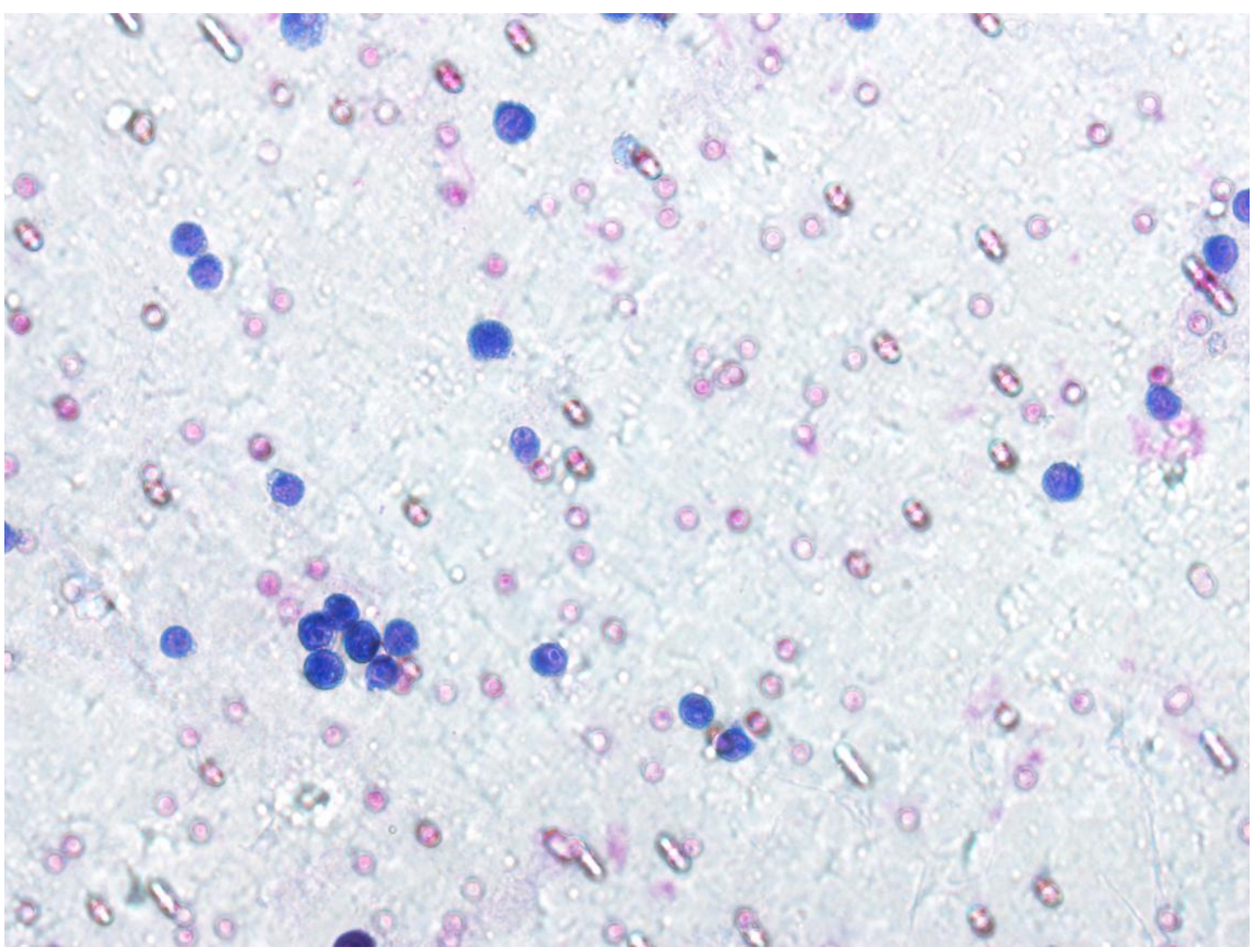

(c)*si-3*#(scale bar = 50 μm)

3. n=3

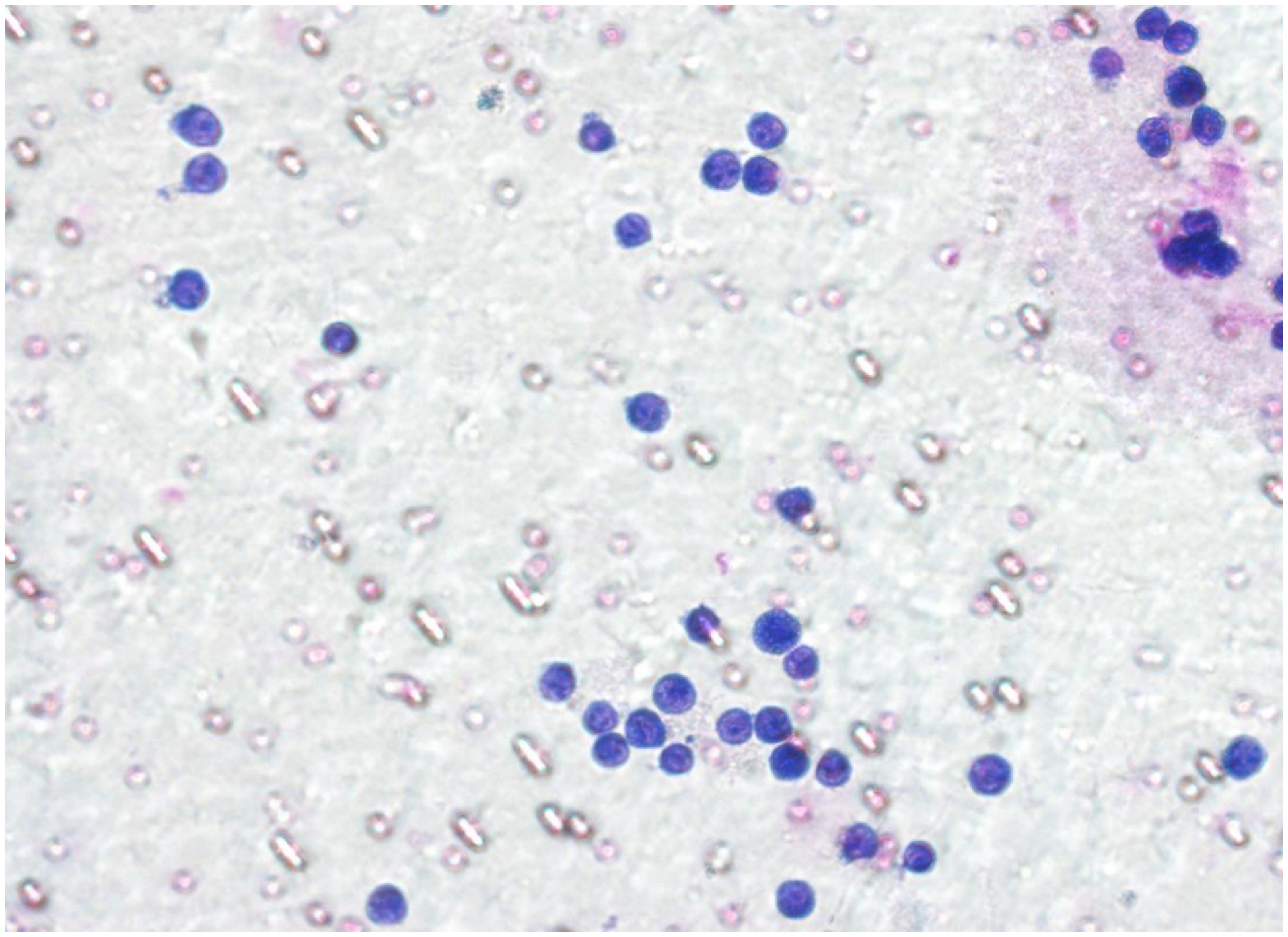

(a)*si-NC*(scale bar = 50 μm)

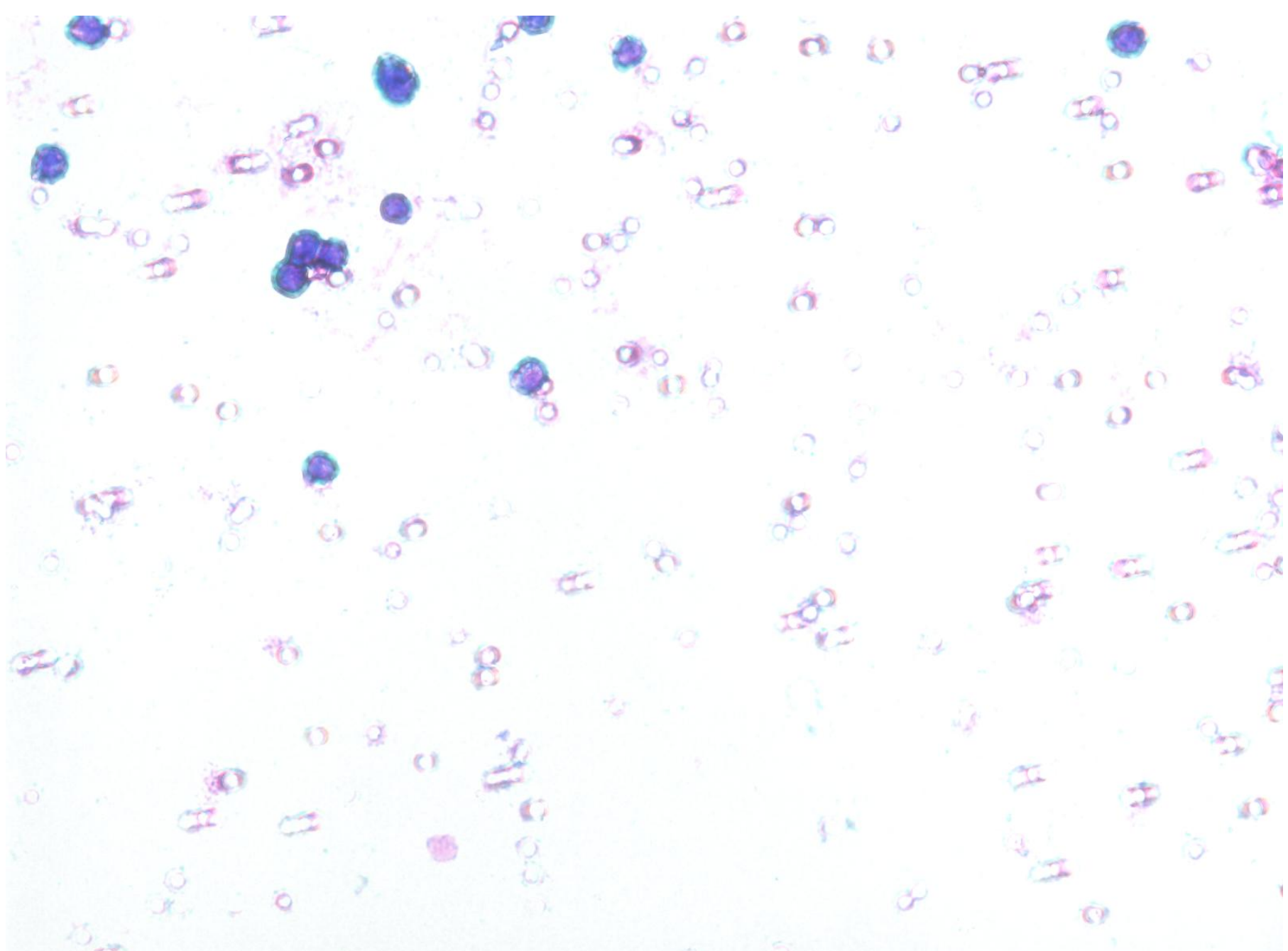

(b)*si-1#*(scale bar = 50μm)

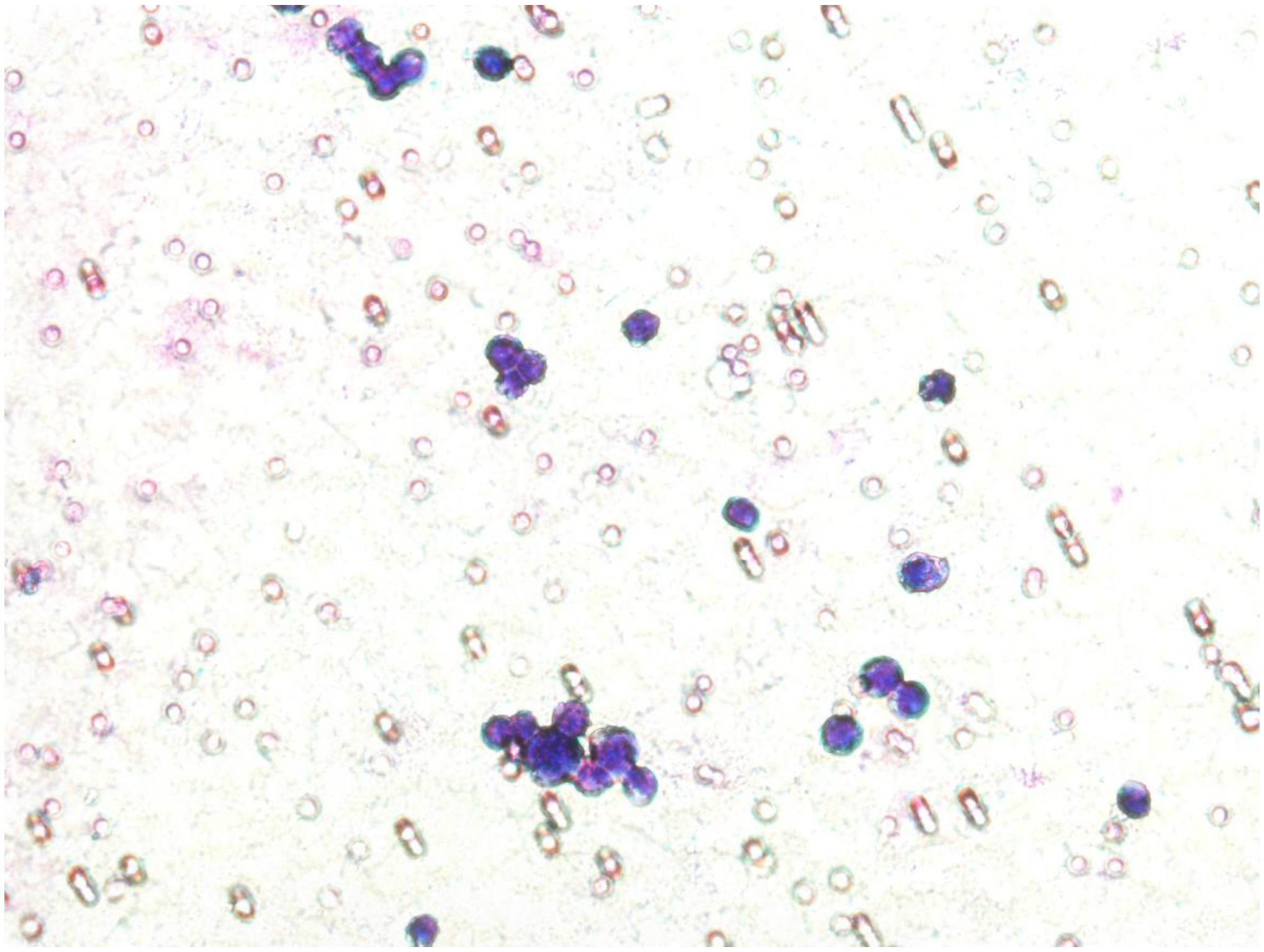

(c)*si-3*#(scale bar = 50 μm)
