# Supplementary material for: Dual relevance of EIF2S1 in acute myeloid leukemia: linking poor prognosis to immune-metabolic states and leukemic cell fitness
Source: Front Oncol. 2026 Aug 5;16:1922910. doi: 10.3389/fonc.2026.1922910 (PMC13485781; doi:10.3389/fonc.2026.1922910)
Supplement: Supplementary file 2 [file DataSheet2.pdf]

Dual Relevance of *EIF2S1* in Acute Myeloid Leukemia: Linking Poor Prognosis to Immune-Metabolic States and Leukemic Cell Fitness

(Manuscript ID: 1922910)

Supplementary Information

Supplementary Method 1.

Independent validation of *EIF2S1* expression and prognosis

EIF2S1 expression was independently evaluated in GSE30029 using normal bone marrow $CD34^+$ cells (n = 31) and AML $CD34^+$ cells (n = 46). Group differences were assessed using a two-sided Wilcoxon rank-sum test. In the TCGA-LAML cohort, *EIF2S1* expression was standardized and analyzed as a continuous variable in univariable and multivariable Cox proportional hazards models. The multivariable model included sex, age, white blood cell count, cytogenetic risk, FLT3 mutation status, and NPM1 mutation status. External prognostic evaluation was performed in the Beat AML 2.0 cohort using one baseline sample per patient. Kaplan–Meier analysis used the cohort-specific median *EIF2S1* expression as the cutoff. Continuous *EIF2S1* expression was analyzed by Cox regression, with hazard ratios reported per one-standard-deviation increase.

Supplementary Methods 2.

Sensitivity analysis according to FAB subtype

To evaluate the potential influence of monocytic differentiation on the immune-related findings, patients with available French–American–British

classification were categorized as FAB M4/M5 or non-M4/M5. M4, M4Eo, M5, M5a, and M5b cases were classified as M4/M5, whereas the remaining annotated FAB subtypes were classified as non-M4/M5. Cases with missing FAB information were excluded from the sensitivity analysis. *EIF2S1* expression was compared between the two groups using a two-sided Wilcoxon rank-sum test. After excluding FAB M4/M5 cases, Spearman correlation analyses between continuous *EIF2S1* expression and ssGSEA-derived signature scores or quanTIseq-estimated cell proportions were repeated. p values were adjusted separately for the ssGSEA and quanTIseq analyses using the Benjamini-Hochberg method. For high- and low-expression comparisons, the median *EIF2S1* value derived from the original full cohort was retained as the cutoff.

Supplementary Figure S1.

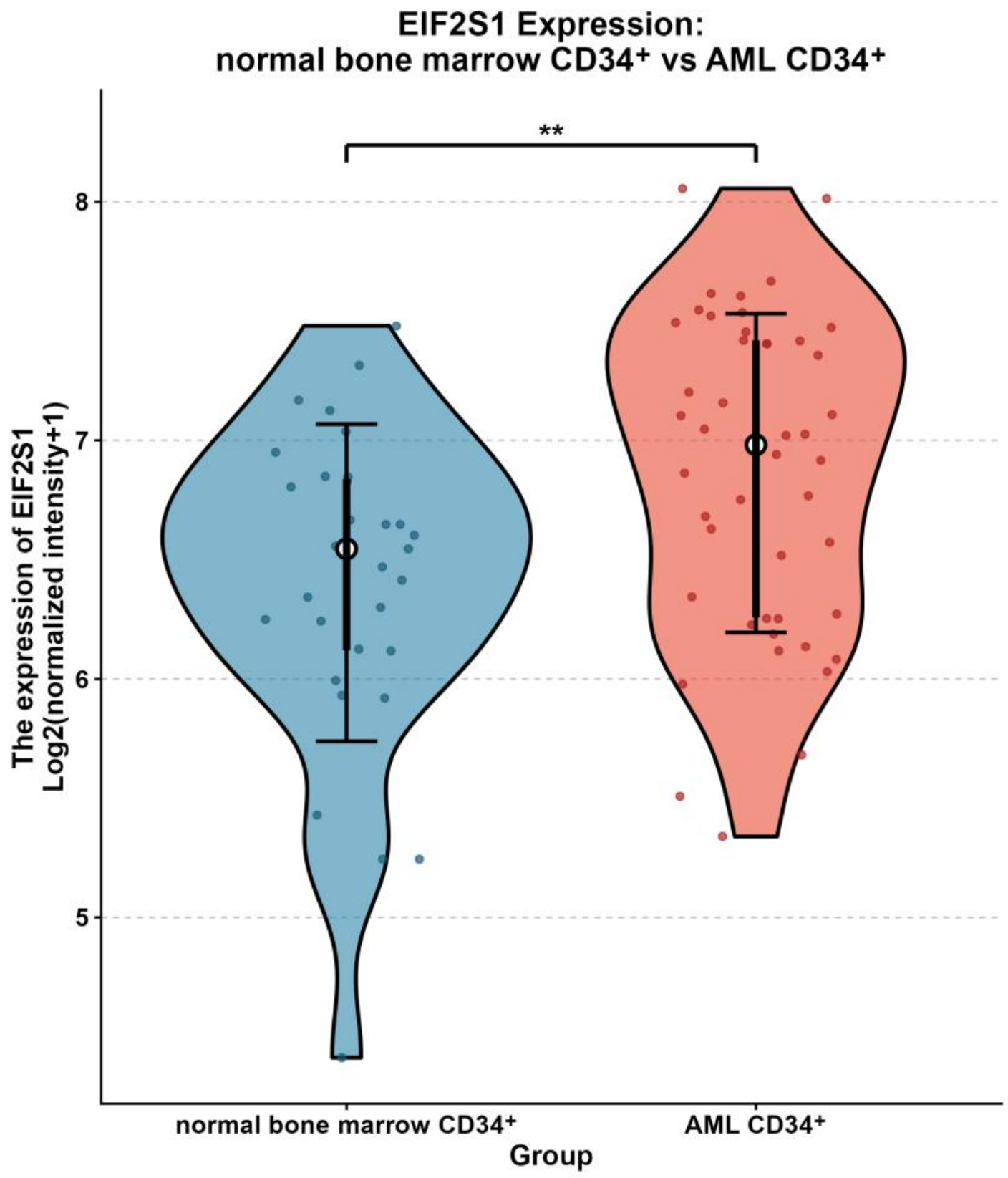

Supplementary Figure S1. External validation of *EIF2S1* expression in the GSE30029 cohort. *EIF2S1* expression was compared between normal $CD34^+$ cells (n = 31) and AML $CD34^+$ cells (n = 46) using a two-sided Wilcoxon rank-sum test ($p = 0.005913$).

Supplementary Figure S2.

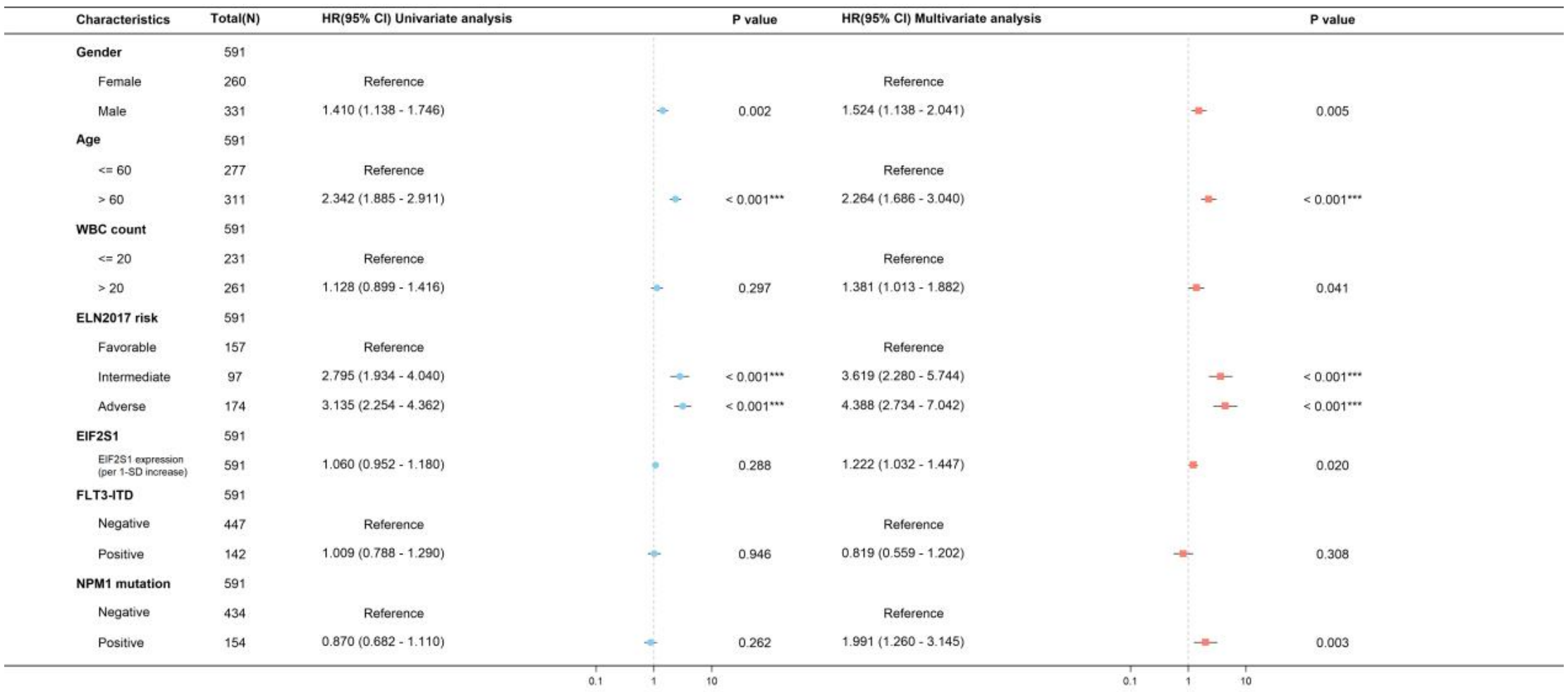

Characteristics	Total(N)	HR(95% CI) Univariate analysis	P value	HR(95% CI) Multivariate analysis	P value
**Gender**	591				
Female	260	Reference		Reference	
Male	331	1.410 (1.138 - 1.746)	0.002	1.524 (1.138 - 2.041)	0.005
**Age**	591				
<= 60	277	Reference		Reference	
> 60	311	2.342 (1.885 - 2.911)	< 0.001***	2.264 (1.686 - 3.040)	< 0.001***
**WBC count**	591				
<= 20	231	Reference		Reference	
> 20	261	1.128 (0.899 - 1.416)	0.297	1.381 (1.013 - 1.882)	0.041
**ELN2017 risk**	591				
Favorable	157	Reference		Reference	
Intermediate	97	2.795 (1.934 - 4.040)	< 0.001***	3.619 (2.280 - 5.744)	< 0.001***
Adverse	174	3.135 (2.254 - 4.362)	< 0.001***	4.388 (2.734 - 7.042)	< 0.001***
**EIF2S1**	591				
EIF2S1 expression (per 1-SD increase)	591	1.060 (0.952 - 1.180)	0.288	1.222 (1.032 - 1.447)	0.020
**FLT3-ITD**	591				
Negative	447	Reference		Reference	
Positive	142	1.009 (0.788 - 1.290)	0.946	0.819 (0.559 - 1.202)	0.308
**NPM1 mutation**	591				
Negative	434	Reference		Reference	
Positive	154	0.870 (0.682 - 1.110)	0.262	1.991 (1.260 - 3.145)	0.003

Supplementary Figure S2. Multivariate Cox regression analysis of *EIF2S1* in the BeatAML2 cohort. Forest plot showing univariate and multivariate Cox regression analyses of overall survival in the BeatAML2 cohort. *EIF2S1* expression was analyzed as a continuous variable per 1-standard-deviation increase. The multivariate model was adjusted for sex, age, WBC count, ELN2017 risk classification, FLT3-ITD status, and NPM1 mutation status. Hazard ratios (HRs), 95% confidence intervals (CIs), and *p* values are shown. A HR greater than 1 indicates increased risk of death.

Supplementary Figure S3.

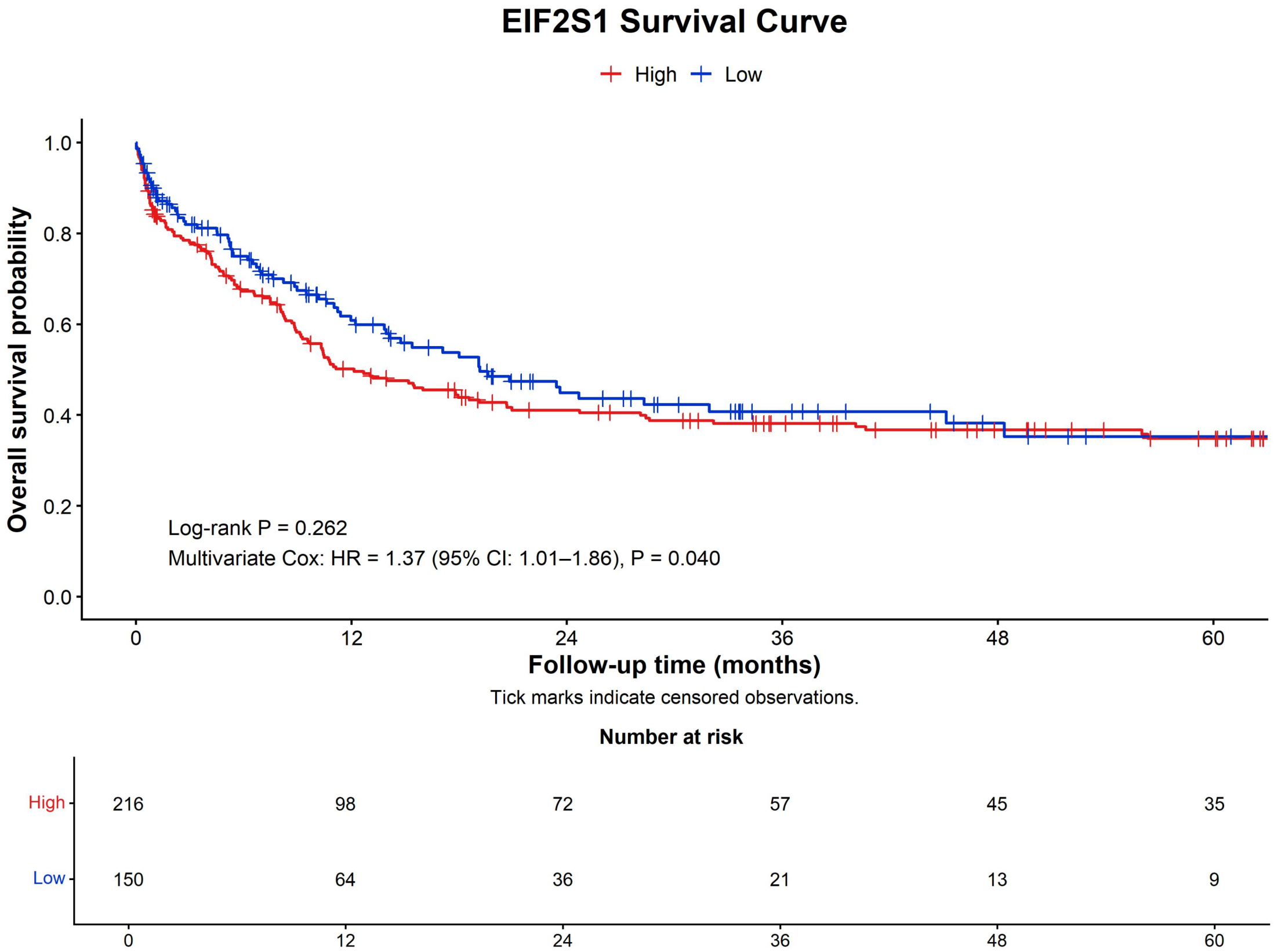

Supplementary Figure S3. External evaluation of the prognostic association of EIF2S1 in the Beat AML 2.0 cohort. Kaplan-Meier survival curves of AML patients in the BeatAML2 cohort stratified by median *EIF2S1* expression. Patients were divided into high- and low-expression groups according to the median *EIF2S1* expression level. Tick marks indicate censored observations, and the number of patients at risk is shown below the plot. The log-rank test was used to compare unadjusted survival curves. Multivariate Cox regression was performed adjusting for sex, age, WBC count, ELN2017 risk classification, FLT3-ITD status, and NPM1 mutation status.

Supplementary Figure S4.

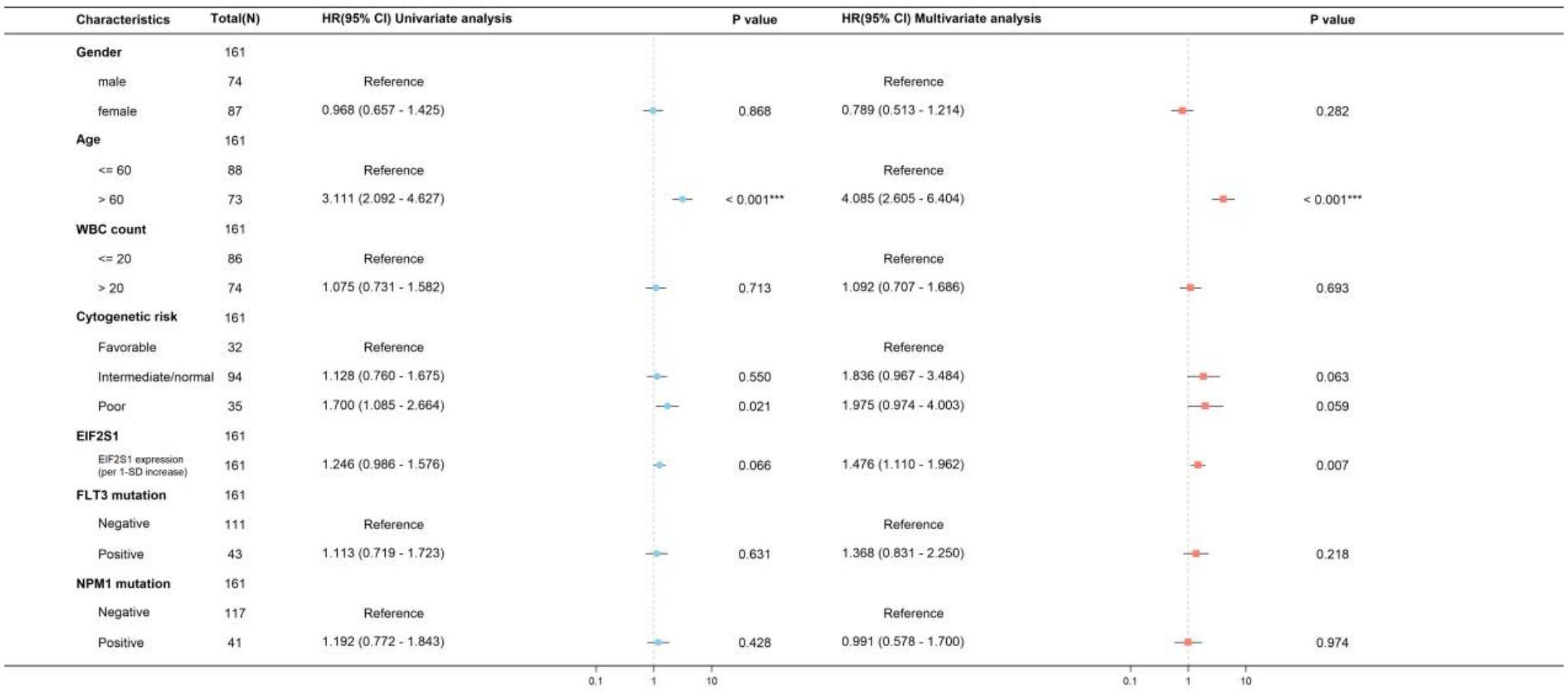

Characteristics	Total(N)	HR(95% CI) Univariate analysis	P value	HR(95% CI) Multivariate analysis	P value
**Gender**	161				
male	74	Reference		Reference	
female	87	0.968 (0.657 - 1.425)	0.868	0.789 (0.513 - 1.214)	0.282
**Age**	161				
<= 60	88	Reference		Reference	
> 60	73	3.111 (2.092 - 4.627)	< 0.001***	4.085 (2.605 - 6.404)	< 0.001***
**WBC count**	161				
<= 20	86	Reference		Reference	
> 20	74	1.075 (0.731 - 1.582)	0.713	1.092 (0.707 - 1.686)	0.693
**Cytogenetic risk**	161				
Favorable	32	Reference		Reference	
Intermediate/normal	94	1.128 (0.760 - 1.675)	0.550	1.836 (0.967 - 3.484)	0.063
Poor	35	1.700 (1.085 - 2.664)	0.021	1.975 (0.974 - 4.003)	0.059
**EIF2S1**	161				
EIF2S1 expression (per 1-SD increase)	161	1.246 (0.986 - 1.576)	0.066	1.476 (1.110 - 1.962)	0.007
**FLT3 mutation**	161				
Negative	111	Reference		Reference	
Positive	43	1.113 (0.719 - 1.723)	0.631	1.368 (0.831 - 2.250)	0.218
**NPM1 mutation**	161				
Negative	117	Reference		Reference	
Positive	41	1.192 (0.772 - 1.843)	0.428	0.991 (0.578 - 1.700)	0.974

Supplementary Figure S4. Continuous-variable Cox proportional hazards analyses of *EIF2S1* in the TCGA-LAML cohort. *EIF2S1* expression was standardized as a Z-score, and HRs are reported per one-standard-deviation increase. The multivariable model included sex, age, WBC count, cytogenetic risk, FLT3 mutation status, NPM1 mutation status, and *EIF2S1* expression. HRs are shown with 95% CIs.

Supplementary Figure S5.

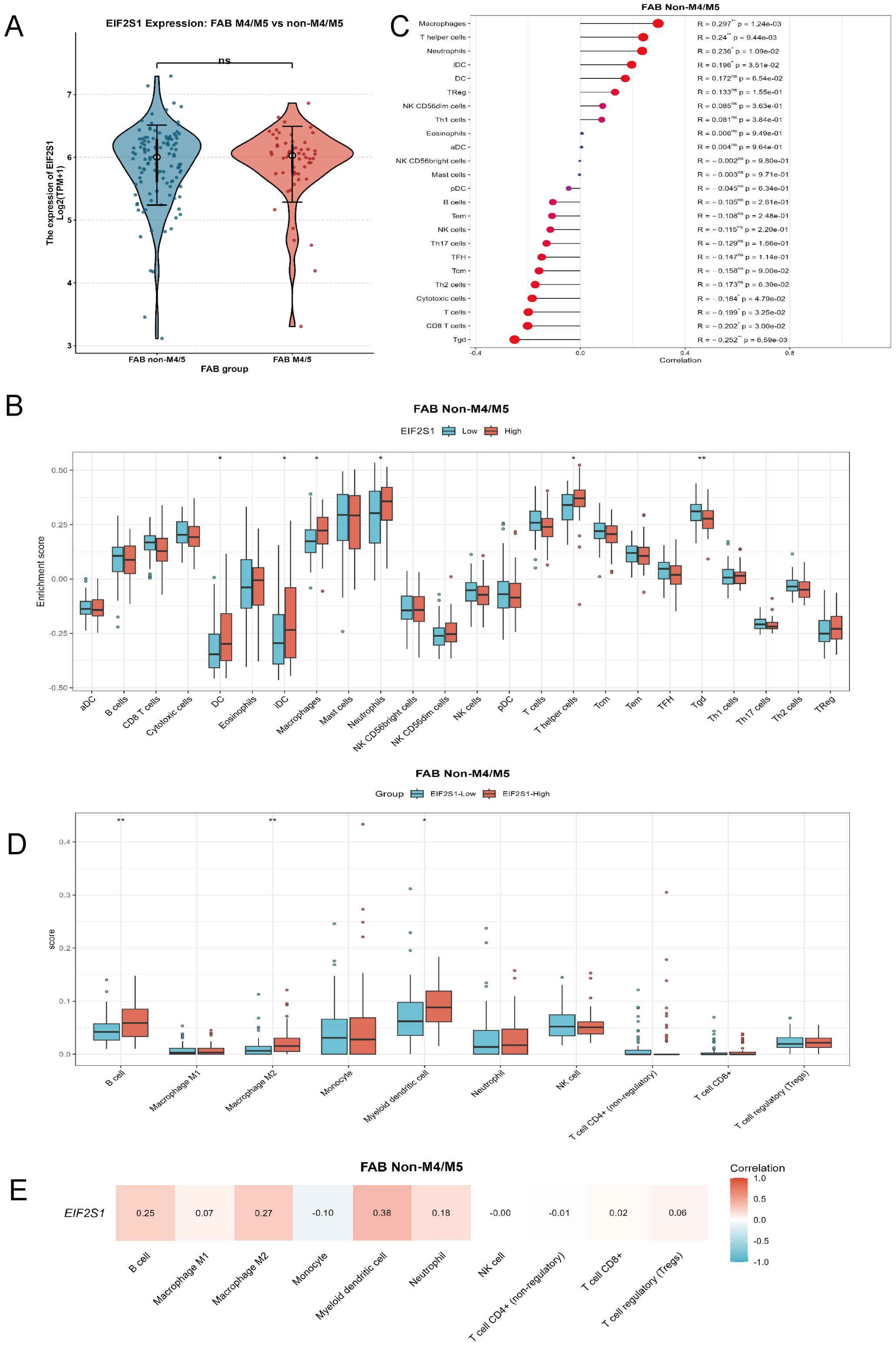

Supplementary Figure S5. Sensitivity analyses of *EIF2S1*-associated immune-related transcriptional features after exclusion of FAB M4/M5 cases.

(A) *EIF2S1* expression in FAB M4/M5 and non-M4/M5 AML cases. (B) ssGSEA-derived immune-cell-associated signature scores in *EIF2S1*-high and *EIF2S1*-low non-M4/M5 cases. (C) Spearman correlations between continuous *EIF2S1* expression and ssGSEA-derived signature scores after exclusion of FAB M4/M5 cases. (D) quanTIseq-estimated immune-cell proportions in *EIF2S1*-high and *EIF2S1*-low non-M4/M5 cases. (E) Spearman correlations between continuous *EIF2S1* expression and quanTIseq-estimated cell proportions after exclusion of FAB M4/M5 cases. p values were adjusted separately for ssGSEA and quanTIseq features using the Benjamini-Hochberg method.

Supplementary Figure S6.

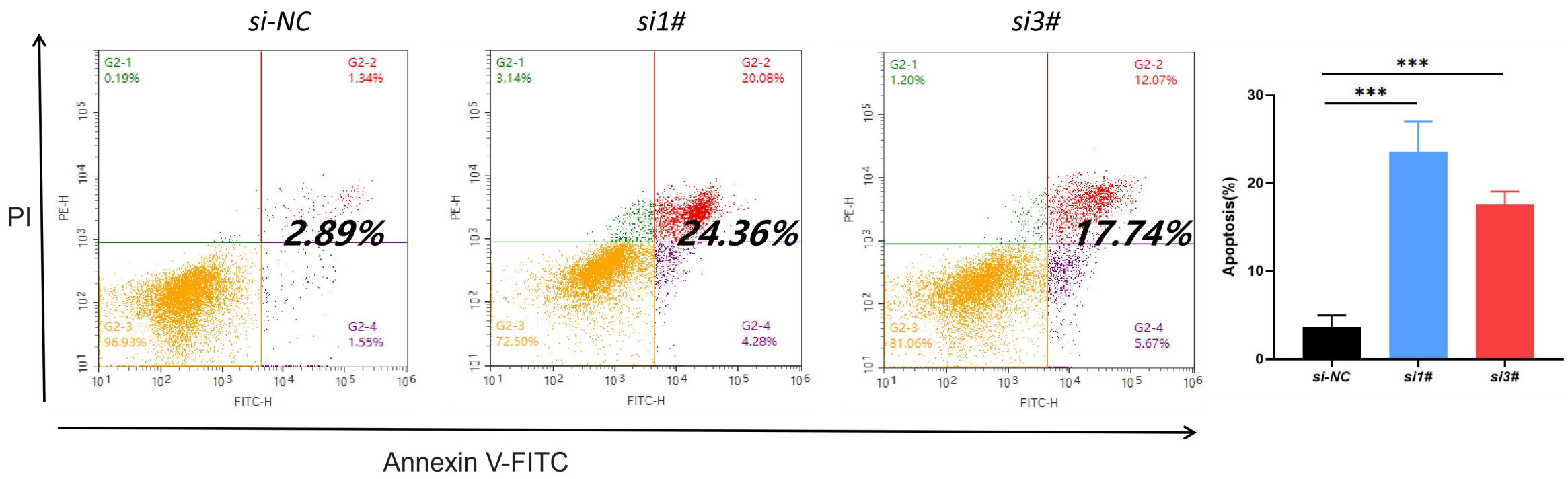

Supplementary Figure S6. Supplementary Figure S6. *EIF2S1* knockdown increases apoptosis in THP-1 cells. THP-1 cells transfected with si-NC, si1#, or si3# were stained using Annexin V－FITC/PI and analyzed by flow cytometry. Data are presented as the mean ± SD from three independent biological experiments.

Supplementary Table 1.

Apoptosis analyses were conducted via flow cytometry

	Early apoptosis (%)	Late apoptosis (%)	Apoptosis(%)
	1.55	1.34	2.89
si-NC	1.56	1.27	2.83
	2.77	2.41	5.18
	4.28	20.08	24.36
si1#	4.43	15.36	19.79
	3.98	22.53	26.51
	4.03	12.13	16.13
si3#	5.67	12.07	17.74
	3.80	15.17	18.97
